# Tumor-associated macrophage-derived exosomal miR21-5p promotes tumor angiogenesis by regulating YAP1/HIF-1α axis in head and neck squamous cell carcinoma

**DOI:** 10.1007/s00018-024-05210-6

**Published:** 2024-04-11

**Authors:** Quan Yan, Jing Liu, Yiding Liu, Zhihao Wen, Dong Jin, Fu Wang, Lu Gao

**Affiliations:** 1 School of Stomatology, Dalian Medical University, No. 9 West Section, Lvshun South Road, Dalian, 116044 People’s Republic of China; 2 Dalian Key Laboratory of Immune and Oral Development & Regeneration, Dalian Medical University, Dalian, People’s Republic of China; 3 The Affiliated Stomatological Hospital of Dalian Medical University, Dalian, People’s Republic of China

**Keywords:** Tumor-associated macrophages, Extracellular vesicles, miR-21-5P, YAP1/HIF-1α axis, Microfluidic chip, Head and neck squamous cell carcinoma

## Abstract

Extracellular vesicles (EVs) have recently received increasing attention as essential mediators of communication between tumor cells and their microenvironments. Tumor-associated macrophages (TAMs) play a proangiogenic role in various tumors, especially head and neck squamous cell carcinoma (HNSCC), and angiogenesis is closely related to tumor growth and metastasis. This research focused on exploring the mechanisms by which EVs derived from TAMs modulate tumor angiogenesis in HNSCC. Our results indicated that TAMs infiltration correlated positively with microvascular density in HNSCC. Then we collected and identified EVs from TAMs. In the microfluidic chip, TAMs derived EVs significantly enhanced the angiogenic potential of pHUVECs and successfully induced the formation of perfusable blood vessels. qPCR and immunofluorescence analyses revealed that EVs from TAMs transferred miR-21-5p to endothelial cells (ECs). And targeting miR-21-5p of TAMs could effectively inhibit TAM-EVs induced angiogenesis. Western blot and tube formation assays showed that miR-21-5p from TAM-EVs downregulated LATS1 and VHL levels but upregulated YAP1 and HIF-1α levels, and the inhibitors of YAP1 and HIF-1α could both reduce the miR-21-5p enhanced angiogenesis in HUVECs. The in vivo experiments further proved that miR-21-5p carried by TAM-EVs promoted the process of tumor angiogenesis via YAP1/HIF-1α axis in HNSCC. Conclusively, TAM-derived EVs transferred miR-21-5p to ECs to target the mRNA of *LATS1* and *VHL*, which inhibited YAP1 phosphorylation and subsequently enhanced YAP1-mediated HIF-1α transcription and reduced VHL-mediated HIF-1α ubiquitination, contributing to angiogenesis in HNSCC. These findings present a novel regulatory mechanism of tumor angiogenesis, and miR-21-5p/YAP1/HIF-1α might be a potential therapeutic target for HNSCC.

**Supplementary Information:**

The online version contains supplementary material available at 10.1007/s00018-024-05210-6.

## Backgroud

Head and neck squamous cell carcinoma (HNSCC) is the most common malignant tumor of the head and neck region, accounting for over 90% of all cases [1]. Despite ongoing advancements in surgical techniques and adjuvant therapies, the 5-year survival rate has not improved significantly in patients with HNSCC having advanced tumors and recurrences. Angiogenesis, the generation of new blood vessels from existing blood vessels to provide nutrients and oxygen to solid tumors, is essential for tumor metastasis [2]. Multiple tumor-associated angiogenic signals are activated during the development of HNSCC, forming new blood vessels in the tumor microenvironment (TME). Therefore, identifying potential targets for tumor-associated angiogenesis is crucial for the treatment of HNSCC.

Tumor-associated macrophages (TAMs) represent the predominant and indispensable immune cell population in the TME, comprising approximately 15–20% of the total mass of solid tumors [3, 4]. Recent research has revealed that TAMs coexist with diverse macrophage subtypes, whereas at advanced tumor stages, TAMs display a predominant M2 macrophage phenotype [5]. Accumulating evidence supports that TAM infiltration is associated with the upregulation of tumor microvessel density (MVD), thereby facilitating the process of tumor hematogenous metastasis [6]. TAMs produce chemokines that promote the proliferation of vascular endothelial cells (ECs), degrade the vascular basement membrane, and induce ECs to migrate into tumors, forming and maturing new microvessels [7]. Therefore, TAMs are key players in tumor-associated angiogenesis and activate a cascade amplification response in this process.

Bidirectional communication between tumor angiogenesis and the TME is critical for sustained tumor growth. Recent studies have shown that tumor-derived exosomes and virus-sized vesicles, which circulate freely in body fluids and accumulate in the interstitium of tumor tissues, are novel factors in angiogenesis [8]. Exosomes are a type of extracellular vesicle (EV) known as small extracellular vesicles (sEV) [9]. sEV collectively refers to membrane-bound small vesicles secreted by cells. These vesicles are crucial for transporting various genetic materials, including proteins and nucleic acids, and mediating intercellular signaling and communication in the TME [10]. In HNSCC, Snail enhances the transcription of miR-21, which is subsequently sorted into sEV, promoting the polarization of M0 towards M2 macrophages [11]. Emerging evidence from recent studies has revealed the essential involvement of TAM-sEV in hematogenous cancer metastasis by transporting diverse signaling molecules [12–14]. TAM infiltration is prominently observed in hypoxic regions, particularly within necrotic tissues [15]. Hypoxia-inducible factor 1-alpha (HIF-1α) can directly regulate the expression of vascular endothelial growth factor (VEGF) at the gene level [16]. Therefore, a close correlation has been observed between TAMs and HIF-1α in the development of tumor-associated angiogenesis. HNSCC is an inflammatory tumor that infiltrates a large number of macrophages [17]. However, the mechanism by which TAM-sEV promotes tumor angiogenesis remains unclear.

An ideal research model for studying angiogenesis should closely mimic the in vivo microenvironment while providing reliable evaluation parameters to assess angiogenesis outcomes. Microfluidic chips are an optimal choice for studying angiogenesis because they can simulate the four essential in vivo conditions of angiogenesis on a single microscale platform, including controlling interstitial flow, inducing factors, intercellular interactions, and three-dimensional tissue scaffolds [18]. These chips effectively replicate the intricate microenvironment, providing a compact and versatile platform for investigating the complexities of angiogenesis, making them invaluable in angiogenesis research.

This study employed microfluidic chips and in vivo experiments to investigate novel mechanisms underlying angiogenesis in HNSCC. Specifically, we showed that the intercellular signaling between TAMs and ECs is mediated by the transfer of miR-21-5p via TAM-sEV. Additionally, the mechanism of miR-21-5p promoting HNSCC angiogenesis involving the YAP1/HIF-1α axis was unveiled. Our findings highlight an important signaling communication of tumor angiogenesis and provide a potential target for HNSCC treatment.

## Methods

### Clinical samples

Thirty HNSCC tissue samples were collected, with complete case information, from the Pathology Department of the First Affiliated Hospital of Dalian Medical University (Dalian, China) between January 2017 and December 2020. None of the patients included in the study received preoperative radiotherapy or chemotherapy, and all provided written informed consent. The expression analysis of 520 patients with head and neck cancer (data sourced from The Cancer Genome Atlas, TCGA, www.cancer.gov/) was conducted using the online tool Tumor IMmune Estimation Resource (TIMER, https://cistrome.shinyapps.io/timer/). This study was approved by the Ethics Committee of the Dalian Medical University (2021002).

### Cell culture

CAL27, SCC25 (tongue squamous carcinoma cell line), and human umbilical vein endothelial cells (HUVECs) were purchased from the American Type Culture Collection (ATCC, Manassas, VA, USA). The human myeloid leukemia mononuclear cell (THP-1) line was obtained from Cellcook (Guangzhou, China). CAL27 and SCC25 cells were often used as typical tumor cells to study of tumor-associated macrophages [19] and cultured in high-glucose Dulbecco’s modified Eagle medium (DMEM; Gibco, Thermo Fisher Scientific, Waltham, MA, USA) supplemented with 10% fetal bovine serum (FBS; Gibco). THP-1 cells were cultured in RPMI 1640 medium (Hyclone, Thermo Fisher Scientific) supplemented with 10% FBS. HUVECs were cultured in EC medium (ECM; ScienCell, Carlsbad, CA, USA) containing 5% FBS and 1% endothelial cell growth supplement (ScienCell). Pulmonary HUVECs (pHUVECs) were obtained from ATCC (Manassas, VA, USA). The culture conditions for the pHUVECs were the same as those for the HUVECs. pHUVECs from the 3rd passage to the 8th were used in microfluidic chip experiments. Normal human fibroblasts (NFs) were isolated from patients whose teeth were extracted at Dalian Medical University (Dalian, China, 2022003). Normal gingival tissues were agitated in 10% DMEM/F12 containing collagenase type I (1 mg/mL; Sigma-Aldrich, SCR103, Merck, Germany) for 10 h, and the dissociated tissues were shaken for 5 min. The supernatant was separated, and NFs were collected with DMEM/F12 medium (10% FBS) at 37 ℃ with 5% CO_2_ in a humidified incubator. All the cells were supplemented with 1% penicillin/streptomycin.

### TAM polarization

THP-1 cells were differentiated into M0 macrophages after a 6 h treatment with 100 ng/mL phorbol 12-myristate 13-acetate (MCE, NJ, USA). M0 macrophages were induced using the supernatant of SCC25/CAL27 cells for 24 h and polarized into TAMs. TAM markers (CD163 and CD206) were detected using flow cytometry and western blotting, and mRNA expression of *Arg1* and *Fizz1* was analyzed by reverse transcription quantitative real-time PCR (RT-qPCR).

### Flow cytometry

1 × 10^6^ HUVECs were harvested and washed twice with PBS by centrifugation at 350–500×g for 5 min. After resuspending with 100 µL of PBS, the cells were incubated with the primary antibody including anti-CD206 (10 μL, Proteintech, APC-65155, Wuhan, China) and anti-CD163 (5 μL, Proteintech, CL488-65169) for 40 min at 4 °C in the dark. Then the cells were washed with PBS for 5 min and the supernatant was discarded. A flow cytometer (FACSVerse, BD Biosciences, USA) were used to detect the cells and the data was analyzed by the software Flowlo 10.8.

### sEV collection, isolation, and identification

When the polarization of M0/TAMs was achieved, the culture medium was removed, and M0/TAMs were washed thrice with PBS. Complete medium comprising 10% FBS without sEV was added to M0/TAMs. After 48 h, culture supernatants of M0/TAMs were collected. We used the ultracentrifugation method to extract M0/TAM-sEV. Briefly, the supernatants were subjected to various centrifugation steps. First, the supernatant was centrifuged at 500×*g* for 20 min at room temperature to remove residual cells. Subsequently, centrifugation at 2500×*g* and 4 °C for 30 min was performed to eliminate cell debris. Then, centrifugation at 12,000×*g* for 45 min was performed to remove large vesicles. Finally, the supernatant was ultracentrifuged at 120,000×*g* for 90 min to enrich the sEV. The pellet was rinsed with 20 mL PBS and ultracentrifuged at 120,000×*g* and 4 °C for 90 min to purify the sEV. For the identification of EVs after purification, three methods were used. The diameter of the sEV was measured using high-sensitivity flow cytometry for nanoparticle tracking analysis (NTA; Nanofcm, Fujian, China). Meanwhile, the M0/TAM-sEV were deposited onto copper grids and negatively stained with 0.2 M phosphotungstic acid for observation under transmission electron microscopy (TEM, JEM-2000EX; JEOL, Tokyo, Japan). Western blot was used to detect the expression of sEV marker proteins, including positive (anti-TSG101, 1:1000, Abcam, ab125011, Cambridge, UK; anti-CD63, 1:1000, Abcam, ab134045; anti-CD9, 1:1000, Abcam, ab263019) and negative (anti-Calnexin; 1:1000, Abcam, ab133615) markers. The pellet containing sEV in 100 μL PBS was stored at −80 °C for further use.

### sEV internalization and delivery assays

M0/TAM-sEV labeled with PKH67 (Thermo Fisher Scientific) were incubated with HUVECs for 6 h. To visualize the HUVECs, DAPI (Solarbio, C0065, Beijing, China) was used to stain the nucleus, and rhodamine-labeled phalloidin (Yeasen, 40734ES75, Shanghai, China) was used to label the cell cytoskeleton. The M0/TAM-sEV were detected using a fluorescence microscope (Olympus, Tokyo, Japan). Transwell chambers (Corning, New York, NY, USA) were used to study the intercellular transfer of miRNAs. TAMs were transfected with FAM mimic/NC and seeded in the upper chamber. HUVECs were seeded in the lower chamber, separated by a 0.4 μm porous membrane. The co-culture was maintained for 48 h. The nuclei of HUVECs were stained with DAPI and observed under a fluorescence microscope to detect the PKH67 (green) fluorescence signal.

### miRNA mimic transfection

HUVECs (1 × 10^5^) were seeded in a 6-well plate, as described previously [20], and Lipofectamine™ 3000 (Thermo Fisher, L3000001) was used to transfect the cells with 10 nM miRNA inhibitor, miRNA mimic, NC inhibitor, and NC mimic (GenePharma, B03001, B02001, B04003, B04002, Shanghai, China). After a 6 h transfection incubation, the culture medium was replaced with complete ECM for 24 h for subsequent experiments.

### Tube formation assay

After 96-well plates (BD, NJ, USA) were coated with Matrigel, HUVECs (2 × 10^4^ cells/well), they were treated with or without M0/TAM-sEV (10 μg/well) for 6 h. Tube-like structures in each well were observed under a light microscope as described previously [21]. To investigate the role of miRNAs in tube formation, HUVECs were transfected with miR-21 mimic or inhibitor for 24 h. The harvested HUVECs were resuspended and seeded in 96-well plates coated with Matrigel for 6 h. Tube formation was observed under a light microscope.

### RNA extraction and RT-qPCR

Total RNA was extracted using TRIzol reagent (Vazyme, R411-01, Nanjing, China), and the total RNA of sEV was extracted using the MolPure^®^ Serum/Plasma miRNA Kit (Yeasen, 19332ES50) as described previously [22]. Briefly, total RNA (1 μg) was reverse transcribed using HiScript II Reverse Transcriptase (Vazyme, R201-01). RT-qPCR was performed using Talent qPCR PreMix (SYBR Green, TIANGEN, FP209, Beijing, China) on a Thermal Cycler Dice Real-Time System (Bio-Rad CFX96 Touch, Hercules, CA, USA). The primer sequences are listed in Supplemental Table 1.

### Western blot

Total protein was extracted by RIPA lysis buffer mixed with PMSF (Solarbio). A BCA protein assay kit was used to determine the protein concentration (Beyotime, P0009, Shanghai, China). Proteins (20 µg) were separated by sodium dodecyl sulfate–polyacrylamide gel electrophoresis (8% for CD206, CD163, CD68, HIF1α and LATS1, 10% for TSG101, CD63, Calnexin, pYAP1, YAP1 and GAPDH, and 12% for CD9 and VHL) and transferred to a PVDF membrane (Millipore Corporation, MA, USA). After membranes were blocked with 5% nonfat milk (Solarbio) at room temperature, the following primary antibodies were used to incubate membranes overnight at 4 °C (anti-CD68, 1:500, 28058-1-AP, Proteintech; anti-CD206, 1:600, 60143-1-Ig, Proteintech; anti-CD163, 1:100, sc-20066, SCBT, CA, USA; anti-VHL40, 1:200, sc-135657, SCBT; anti-LATS1, 1:5000, 17049-1-AP, Proteintech; anti-YAP1, 1:4000, 13584-1-AP, Proteintech; anti-pYAP1, 1:4000, T55743, Abmart, Shanghai, China; anti-HIF-1α, 1:500, ab308433, Abcam; anti-GAPDH, 1:8000, ab8245, Abcam). The membranes were then incubated with horseradish peroxidase-conjugated secondary antibodies (1:8000, ab205719/ab205718, Abcam) for 1 h, and the protein bands were detected using an ECL detection system (1705061, Bio-Rad).

### Immunohistochemistry and immunofluorescence

Paraffin-embedded tissue sections (4-μm-thick) were treated with xylene and rehydrated in gradient alcohol. After treatment with 3% hydrogen peroxide for 20 min and antigen retrieval using sodium citrate buffer (G1202-250ML, Servicebio, Wuhan, China), the sections were blocked with 10% nonimmune goat serum (Gibco, Thermo Fisher) for 1 h. Next, the sections were incubated with the following primary antibodies overnight at 4 °C (anti-CD31, 1:50, ab28364, Abcam; anti-CD206, 1:500, 60143-1-Ig, Proteintech; anti-CD163, 1:50, sc-20066, SCBT). Antibody binding was detected using a peroxidase secondary antibody (1:500, Abcam), followed by counterstaining with hematoxylin. Finally, the integrated optical density (IOD) values of the brown staining were measured using Image-Pro Plus 6.0. The IOD/area ratio was calculated to obtain semi-quantitative values of protein expression.

For immunofluorescence staining, 0.5% Triton-X was used to permeabilize the sections. Next, we blocked the tissues with 10% nonimmune goat serum before incubating them overnight at 4 °C with the following primary antibodies: anti-CD31 (1:20, ab28364, Abcam), anti-CD206 (1:500, 60143-1-Ig, Proteintech), and anti-CD163 (1:200, sc-20066, SCBT). Sections were then incubated with Dylight-488 (1:200; A23220, Abbkine, CA, USA) or Dlight-549 (1:200, A23320, Abbkine) at room temperature for 1 h. Finally, we stained the sections with DAPI and observed them under a fluorescence microscope.

### Microfluidic chip assay

The microfluidic chip comprises five channels (C1-C5) with a width of 800 µm. Each channel is separated by pillars and has an inlet and outlet. The interconnected segments between the five channels were all 5.5 mm in length. Channels C1/C2 and C4/C5 were divided by pentagonal microcolumns with a width of 200 µm and a spacing of 90 µm. Channels C2/C3 and C3/C4 were divided by hexagonal microcolumns with a width of 200 µm and a spacing of 90 µm.

The silicon wafer was coated with a SU8-3035 negative photoresist (17,020,067 Microchem Corp., NH, USA) to a thickness of 120 µm. Desired design patterns were created using photolithography. Polydimethylsiloxane (PDMS; Dow Corning Corp, MI, USA) was poured into the prepared photomask and subjected to 30 min of vacuuming to remove air. After the mold was cured at 80 ℃ for 45 min, the PDMS was peeled off from the wafer and trimmed to the desired size. Outlet 2, Outlet 4, Inlet 2, and Inlet 4 were punched using a 4 mm hole punch, whereas Outlet 1, Outlet 3, Outlet 5, Inlet 1, Inlet 3, and Inlet 5 were punched using a 2 mm hole punch. To bond the PDMS membranes to petri dishes (Corning, NY, USA), the membranes were treated with plasma for 1 min. The assembly was placed in a drying oven and baked at 80 ℃ for 12 h, followed by a 12 h UV sterilization.

Fibrinogen (Sigma Aldrich) was dissolved in a 0.9% sodium chloride solution to a concentration of 10 mg/mL. D-PBS (Hyclone) was used to dilute the fibrinogen solution to 4 mg/mL, and then antipain (0.45 TIU/mL, EI3, Sigma-Aldrich) was added. The fibrinogen solution was then filtered through a sterile filter. Thrombin (1 U/mL, 10602400001, Sigma-Aldrich) was mixed with a sterile-filtered fibrinogen solution to induce fibrinogen clot formation. After the fibrinogen mixture was introduced into channels C3 and C5 and mixed with digested NF, the mixture was added to channel C1, where the cell density was adjusted to 2 × 10^6^ cells/mL. The microfluidic chip was kept at 37 ℃ for 15 min to allow fibrinogen gelling. After ECM was added to channels C2 and C4 for 24 h, pHUVECs were introduced into channel C4 at a density of 2 × 10^6^ cells/mL. M0/TAM-EVs were added to channel C2, and different ECM volumes were added to channels C2 and C4 to create a 1 mm H_2_O liquid-level difference, forming an interstitial flow. To maintain the stability of the interstitial flow, the medium was changed every 12 h and observed under a microscope every 24 h.

### Single-cell sequencing data source and processing

The scRNA-seq dataset GSE103322 for HNSCC [23], which includes 5902 cells from 18 patients, was downloaded from the Gene Expression Omnibus (GEO) database (www.ncbi.nlm.nih.gov/). The Seurat package in R software was used to create the Seurat object [24]. Genes detected in fewer than three cells and cells expressing fewer than 200 unique genes were excluded from downstream analyses. Subsequently, we calculated the sequencing depth (within 20,000), number of expressed gene types (300–10,000), proportion of red blood cell genes (<3%), and proportion of mitochondrial genes (<10%) for each cell. High-quality cell data were normalized to find 2000 high-variable genes for subsequent analyses. Based on 2000 highly variable genes, principal component analysis (PCA) was applied to reduce the dimensionality of the data, and clustering was conducted to select 30 principal components (PCs), which were visualized using T-distributed neighbor embedding (t-SNE) [25]. The FindNeighbors function from the Seurat package was then employed to identify neighbors for each cell, and the FindClusters function (within a resolution of one) was applied to the cluster cell types based on the 30 PCs. The t-SNE algorithm was used to visualize the cell clustering results. Additionally, we combined the “SingleR” package with existing marker genes for cell annotation [26], resulting in the identification of 10 cell types. Bubble plots were used to display the expression of marker genes in different cell types. The expression of four exosomal marker genes across different subtypes are derived from normalized datasets and visualized using a violin plot constructed with ggplot2 [27].

### Cell–cell interaction analysis

We used CellPhone DB to investigate the interactions between various types of cells [28]. First, we created a Linux subsystem using Ubuntu 22.04.2 and set up a Python 3.6 environment for CellPhone DB. The annotation information and expression matrices of the ten cell types were extracted from the Seurat objects. Cell communication analysis was performed using CellPhone DB 4.0 with 1000 iterations to obtain ligand–receptor pairs involved in the interactions between the 10 cell types. Finally, a heatmap was generated based on the number of ligand–receptor pairs that mediate cell–cell interactions.

### Enrichment analysis and cell pathway activity analysis

We used R software (version 4.1.3) and the FindAllMarkers function of the Seurat package, employing the Wilcoxon test as the statistical method to identify signature genes for each cell type. Using the false discovery rate (FDR), only genes with FDR-adjusted *p* values less than 0.05 were considered significantly dysregulated. In ECs, 1492 signature genes were identified, while 1635 signature genes were identified in macrophages. Enrichment analysis was performed using the clusterProfiler package in R software [29]. Specifically, we conducted Gene Ontology Biological Process (GO.BP, htpps://geneontoloy.org/) and Kyoto Encyclopedia of Genes and Genomes (KEGG, www.genome.jp/kegg/) enrichment analyses separately for 1635 signature genes in macrophages and 1492 signature genes in ECs. Furthermore, to evaluate the pathway activity of VEGF, HIF-1α, and Hippo pathways in all cells, the SCpur package was used to generate an enrichment heatmap [30], visualizing the pathway activity based on the expression levels of pathway-associated genes.

### Matrigel-plug angiogenesis assay

We used the Matrigel (Corning, 354248) injection model to detect angiogenesis in vivo. First, 50 μL of PBS, 50 μL M0-sEV (100 μg) and 50 μL TAM-sEV (100 μg) with or without pre-treatment of antagomir (1 OD; B05001, Genepharma, Shanghai, China) was respectively mixed with 450 μL of Matrigel. The mixture was injected subcutaneously into the ventral region of female BALB/c nude mice (4 weeks old, *n* = 4 per group). After 2 weeks, the mice were sacrificed, and Matrigel plugs were fixed with paraformaldehyde and embedded in paraffin wax. Serial sections were prepared and stained with hematoxylin and eosin (HE) staining and immunohistochemistry. The microvessel area in each sample was quantified using Image-Pro Plus 6.0. All the animal experiments were approved by the Institutional Animal Care and Use Committee of Dalian Medical University (No. AEE23070).

### Tumor xenograft assay

To establish the subcutaneous tumor xenograft model, 4-week-old female BALB/c were randomly assigned into four groups (PBS, M0-sEV, TAM-sEV, TAM-sEV/antagomir), and each mouse was injected subcutaneously in the flanks with 1 × 10^6^ SCC25 cells mixed with 100 μL Matrigel. Then, according to the established four subgroups, we started injecting PBS (50 μL), M0-sEV (50 μL, 100 μg), and TAM-sEV (50 μL, 100 μg) with or without antagomir (1 OD, B05001, Genepharma) every 3 days (six times in total). The volume of the tumor (length × width^2^/2) and weight of the mice were monitored every 2 days throughout the study period. After 1 month, the mice were euthanized, and their tumors were collected. Tumor volumes were calculated using the formula *V* (mm^3^) = *A* × *B*^2^/2 (*A* is the largest diameter and *B* is the perpendicular diameter). Finally, the harvested tumors were fixed in 4% paraformaldehyde at 4 °C and embedded in paraffin for subsequent histochemical staining.

### Statistical analyses

GraphPad Prism (version 7.0) was used for the statistical analysis. The correlation between tumor MVD and TAM markers in tumor tissues was assessed using Pearson’s correlation analysis. To calculate *p* values, we used Student’s *t* test and one-way analysis of variance. Statistical significance was set at *p* < 0.05. We expressed the data as the mean ± SEM of at least three independent experiments.

## Results

### TAMs positively correlated with MVD in HNSCC

**Fig. 1:**
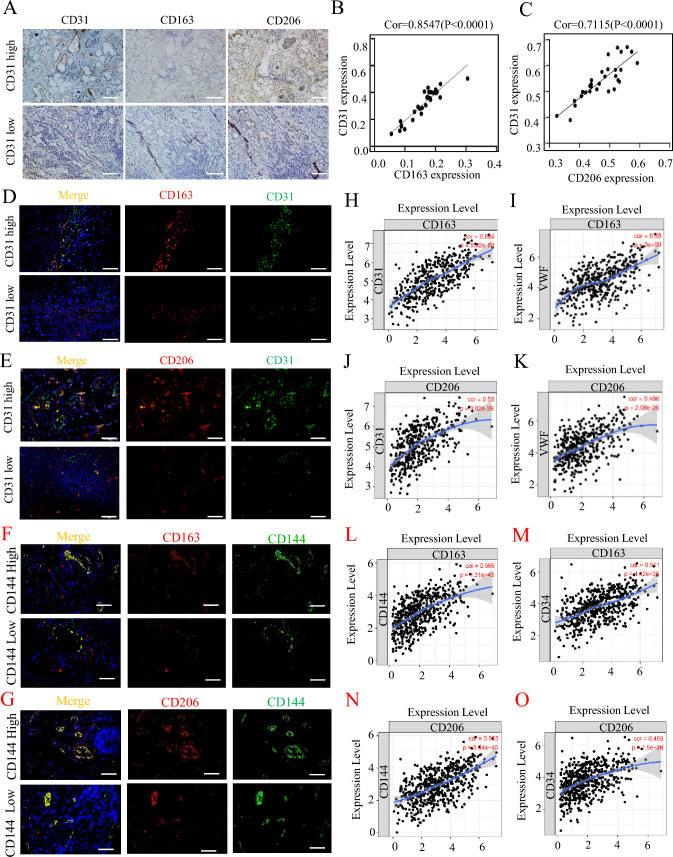
TAMs increase MVD in HNSCC tissues. **A** Representative images of immunohistochemistry (IHC) staining for CD31, CD163 and CD206 in OSCC tissues. Scale bars = 100 μm. **B** Pearson correlation analysis of the mean density of CD163 and MVD (CD31) in human OSCC tissues. **C** Pearson correlation analysis of the mean density of CD206 and MVD (CD31) in human OSCC tissues. **D** Representative images of IF staining for CD163 and CD31 in OSCC tissues, Scale bars = 50 μm. **E** Representative images of IF staining for CD206 and CD31 in OSCC tissues, Scale bars = 50 μm. **F** Representative images of IF staining for CD163 and CD144 in HNSCC tissues, Scale bars = 50 μm. **G** Representative images of IF staining for CD206 and CD144 in HNSCC tissues, Scale bars = 50 μm. **H**,**I** Spearman’s correlation analysis of the mRNA expression profiles of CD163 and CD31, as well as CD163 and VWF in 520 HNSCC patients from TCGA. **J**,**K** Spearman’s correlation analysis of the mRNA expression profiles of CD206 and CD31, as well as CD206 and VWF in 493 HNSCC patients from TCGA. **L**,**M** Spearman’s correlation analysis of the mRNA expression profiles of CD163 and CD144, as well as CD163 and CD34 in 520 HNSCC patients from TCGA. **N**,**O** Spearman’s correlation analysis of the mRNA expression profiles of CD206 and CD144, as well as CD206 and CD34 in 493 HNSCC patients from TCGA

Using 30 HNSCC tissue samples, immunostaining for CD31 was employed to assess MVD, whereas CD163 and CD206 were used to detect the infiltration. The average optical densities of CD31, CD163, and CD206 were quantified to analyze MVD and TAMs. Our results showed a significant increase in MVD in HNSCC tissues with high expression of CD163 and CD206 compared to those with low expression of CD163 and CD206 (Fig. 1A). Pearson’s correlation analysis indicated a significant positive correlation between the infiltration levels and MVD in HNSCC (*p* < 0.0001; Fig. 1B, C). Further, immunofluorescence staining revealed that in HNSCC tissues, CD163 and CD206 expression occurred around CD31 and CD144, and the colocalization of CD163 and CD206 was positively correlated with CD31 and CD144 (Fig. 1D–G). To further corroborate the association between TAMs and MVD, we conducted an extensive analysis using data from The Cancer Genome Atlas (TCGA) comprising 520 cases of head and neck tumors [31]. Expression levels of von Willebrand factor, CD31, CD34 and CD144 mRNA were used as surrogate markers for MVD, whereas CD206 and CD163 mRNA levels were used to assess TAM infiltration. Our findings demonstrated a compelling and statistically significant positive correlation between TAMs and MVD (*p* < 0.0001; Fig. 1H–O). These results suggest that infiltration in HNSCC is associated with tumor angiogenesis.

### TAMs-derived EVs promote angiogenesis of HUVECs in vitro

**Fig. 2:**
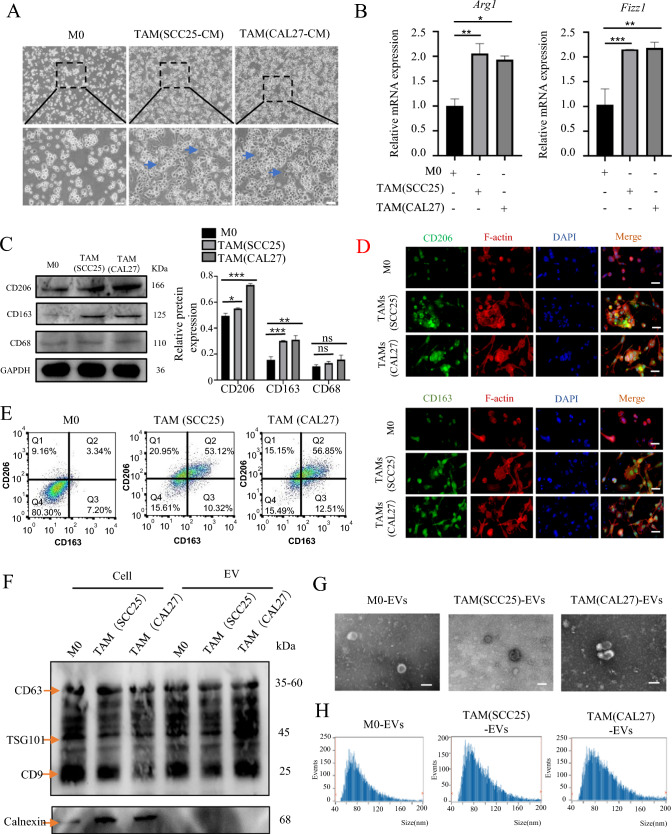
TAMs from HNSCC predominantly exhibit the M2 phenotype. **A** The morphological changes that occur when M0 macrophages were induced into TAMs by SCC25/CAL27-conditioned medium. The blue arrow indicated the emergence of pseudopodia in TAMs. **B** RT-qPCR was used to detect the mRNA levels of M2 phenotype markers (*FIZZ-1*, *ARG-1*) in TAMs. **C** Western blot was used to detect the protein levels of the M2 phenotype markers in TAMs (CD163, CD206). **D** Representative images of IF staining for CD163, CD206 and F-actin in macrophages induced by HNSCC conditioned medium, Scale bars = 50 μm. **E** Expression of classical M2 macrophage markers (CD206 and CD163) was evaluated by flow cytometry. **F** Western blot was performed to detect the expression of positive markers (TSG101, CD63, CD9) and negative marker (Calnexin) in the TAM-EVs. **G** Electron microscopy images of EVs were isolated from M0/TAM-CM. **H** Nanoparticle tracking analysis (NTA) of M0/TAM-sEVs. ns > 0.05, * *p* < 0.05, ** *p* < 0.01, *** *p* < 0.001

**Fig. 3:**
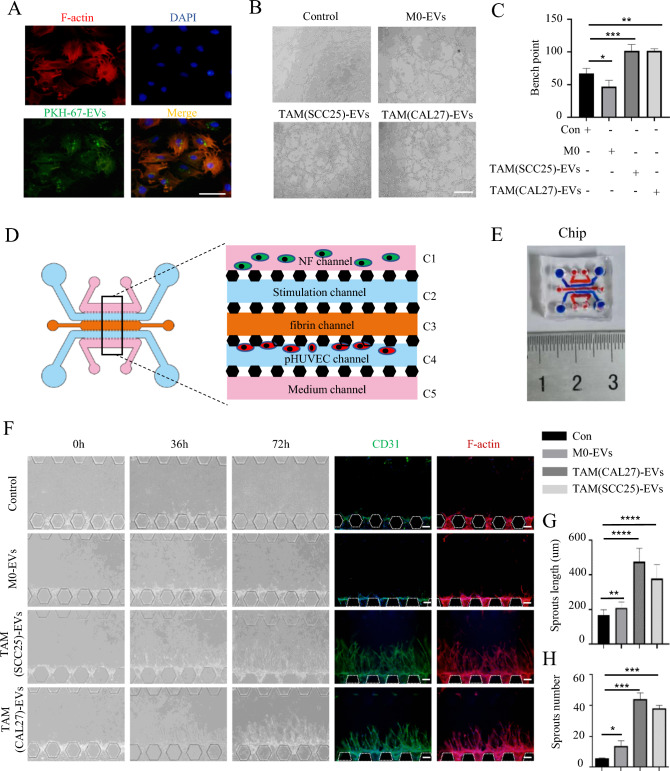
TAMs-EVs promote angiogenesis of HUVECs in vitro. **A** IF image demonstrates that HUVECs have taken up and internalized PKH67-labeled sEVs derived from macrophages, Scale bars = 50 μm. **B** Representative micrographs of tube formation assay induced by M0/TAM-sEVs, Scale bars = 100 μm. **C** The numbers of branch points were calculated by ImageJ. **D** Schematic overview (left) and channel configuration (right) of the microfluidic chip designed for the induction of angiogenesis. **E** Physical diagram of a microfluidic chip compared with a one-yuan Chinese currency coin. **F** Images of vascular sprouts induced by M0/TAM-sEVs, Scale bars = 100 μm. **G**,**H** Quantitative analyses of angiogenesis in terms of average sprout length and number. ns > 0.05, * *p* < 0.05, ** *p* < 0.01, *** *p* < 0.001, **** *p* < 0.0001

To obtain TAMs, we collected SCC25-CM and CAL27-CM. After a 24 h induction of M0 macrophages with SCC25-CM or CAL27-CM, the M0 macrophages underwent a transition from a spherical morphology to flattened TAMs, accompanied by the emergence of pseudopodia (Fig. 2A). Following induction, RT-qPCR analyses of TAMs showed that the expression of M2 macrophage markers (*Arg1*, *Fizz1*) was significantly increased compared to that in the M0 group (Fig. 2B). Western blotting and flow cytometry showed that the expression of CD163 and CD206 in TAMs was higher than in M0 macrophages (Fig. 2C, E). The immunofluorescence results also showed a remarkable enhancement in the expression of CD163 and CD206 in the TAM group (Fig. 2D). These results indicated that TAMs polarized by SCC25-CM and CAL27-CM primarily exhibited M2-like macrophages. It is well known that the exosome is an important pathway of cellular communication. We have demonstrated the correlation between TAMs and MVD, it is then possible that TAMs promote tumor angiogenesis through TAM-EVs. Next, we employed differential centrifugation to isolate and purify EVs from M0/TAM-CM. After lysis, collected M0/TAM-EVs were subjected to western blotting. The results showed that the M0/TAM-EVs were positive for EV markers, such as TSG101, CD63, and CD9, and negative for calnexin (Fig. 2F). The collected M0/TAM-EVs exhibited typical disc-shaped structures, as observed by transmission electron microscopy (Fig. 2G). In addition, we conducted NTA analysis of EVs in the conditioned medium of TAMs at different time points. NTA revealed that the collected EVs at 48 h of M0/TAMs-CM had high purity (Fig. S1A) and fell within the diameter ranging of 40–200 nm, confirming their classification as sEVs (Fig. 2H). Fluorescence microscopy revealed that HUVECs were capable of internalizing macrophage-derived sEVs and localizing them around the nucleus (Fig. 3A). Tube formation assays showed that TAM (SCC25)-sEVs/TAM (CAL27)-sEVs significantly enhanced the tube-forming ability of HUVECs, whereas M0-sEVs had little effect (Fig. 3B, C). In addition, the tube formation indicated that both TAM-CM and TAM-EVs could promote angiogenesis, but TAM-CM without EVs did not have a significant effect on HUVECs, suggesting that EVs in TAM-CM play an important role in promoting tumor angiogenesis (Fig. S1B).

To further investigate the effect of TAM-sEVs on HNSCC angiogenesis, we designed a microfluidic chip to mimic the angiogenic microenvironment in vivo (Fig. 3D). The microfluidic chip allows real-time observation of fluids and cells at the micrometer scale with high throughput and a small volume (Fig. 3E). In the C1 channel, three-dimensional cultured NFs were seeded to maintain the stability of vascular sprouts. The C2 channel was supplemented with vascular induction factors. The C3 channel served as the fibrinogen channel, providing a mesh scaffold for vascular sprouting. pHUVECs were seeded in the C4 channel, and deposited between C3 and C4 channels to simulate the vascular endothelium. The C5 channel served as the culture medium channel, providing nutritional support to the microfluidic chip. According to the previous study [32], after VEGF (50 ng/mL) was added to the C2 channel and pHUVECs were induced for 48 h, the formation of vascular sprouts was evident, confirming the successful modeling of vascular formation in the microfluidic chip (Fig. S2A). Subsequently, we added M0-sEVs and TAM-sEVs to the C2 channel of the microfluidic chip, both of which induced the formation of vascular sprouts in pHUVECs. In comparison, the number of vascular sprouts induced by TAM-EVs was greater, the lengths were more, and the shape was more robust than those induced by M0-sEVs (Fig. 3F–H). After 7 days, TAM-sEVs successfully induced the formation of continuous vessels connecting C4 to the C2 channel in the microfluidic chip (Fig. S2B). These results provide evidence that TAM-sEVs promote vascular formation in pHUVECs and might play a pivotal role in HNSCC angiogenesis.

### TAM-EVs enhanced angiogenesis by upregulating the level of HIF-1α in HUVECs

Single-cell sequencing of head and neck cancer was performed using the previous GEO dataset (GSE103322) [23]. After low-quality cells and genes were filtered out, 5886 cells were used for subsequent analyses (Fig. S3A). We selected 2000 highly variable genes (Fig. S3B) by variance stabilization transformation, and dimensionality reduction was performed using PCA with 30 PCs (Fig. S3C) while accounting for cell cycle effects. After using the t-SNE [25] algorithm for cluster analysis and visualization, the 5886 cells were grouped into 25 distinct clusters (Fig. S3D). Through manual and machine annotation, we identified 10 cell types: epithelial cells, T cells, fibroblasts, macrophages, mast cells, ECs, lymphatic ECs, dendritic cells, B cells, and muscle cells. The marker genes of the 10 cell types are displayed using bubble plots (Fig. S3E, F).

**Fig. 4:**
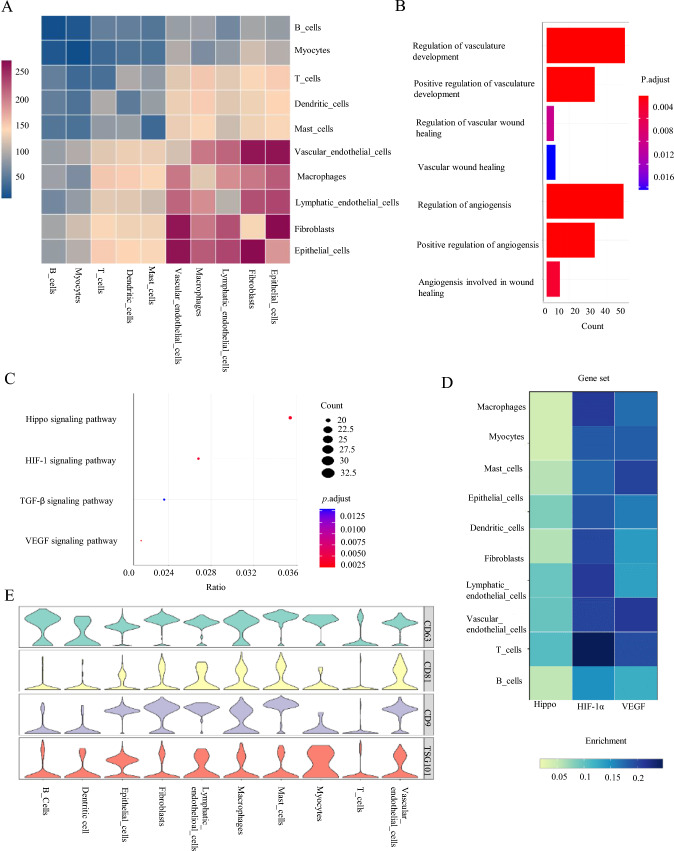
Cell chat and enrichment analysis in TAMs and ECs. **A** Cell phoneDB cell communication analysis was performed on 10 cell types using Python 3.6 environment in Linux subsystem ubuntu 22.04.2. The analysis involved detecting the interactions between cells, identifying ligand-receptor pairs, and the results were presented using a heatmap to visualize the quantity of cell–cell interactions. **B** GO.BP enrichment analysis was conducted on 1634 signature genes in macrophages using the clusterProfile package (R 4.3.0). **C** KEGG enrichment analysis was perform package. **D** Using the Scpubr package in R 4.1.3, the pathway activities of Hippo, HIF-1α, and VEGF was inferred based on the expression levels of pathway genes and visualized using heatmaps. **E** Violin plot illustrating the expression patterns of exosomal markers (CD63, CD81, CD9 and TSG101) across subpopulations in HNSCC

To further explore the cell–cell interactions between TAMs and vascular ECs, we conducted a cell communication analysis using the CellPhone DB [28]. The analysis revealed 203 ligand–receptor pairs interacting between TAMs and ECs, which demonstrated a relatively significant level of interaction by TEM (Fig. 4A). After applying the Wilcoxon test to the “FindAllMarkers” function in the R package, we identified 1635 signature genes for macrophages and 1492 signature genes for ECs (FDR < 0.05). The signature genes were analyzed using GO.BP and KEGG [29]. The results were visualized using bar and bubble plots (Fig. 4B, C). From the enrichment results, we observed that macrophages were associated with functions related to angiogenesis, wound healing, and vascular tissue remodeling. Additionally, Hippo, HIF-1α, and VEGF pathways related to vascular development were significantly enriched in ECs. Subsequently, we used gene expression levels to calculate Hippo, HIF-1α, and VEGF activity for each cell type and visualized it through a heatmap (Fig. 4D). The heatmap shows that ECs and macrophages both exhibited the highest HIF-1α activity among other cell types. The violin diagram shows that in HNSCC tissue, macrophages highly expressed four classic marker genes (CD63, CD9, CD81, TSG101) of exosomes, suggesting that TAM-EVs are highly secreted in the tumor microenvironment (Fig. 4E). Based on these results, we speculate that macrophages may promote angiogenesis in HNSCC by upregulating HIF-1α activity, and TAM-EVs may play an important role in this process.

**Fig. 5:**
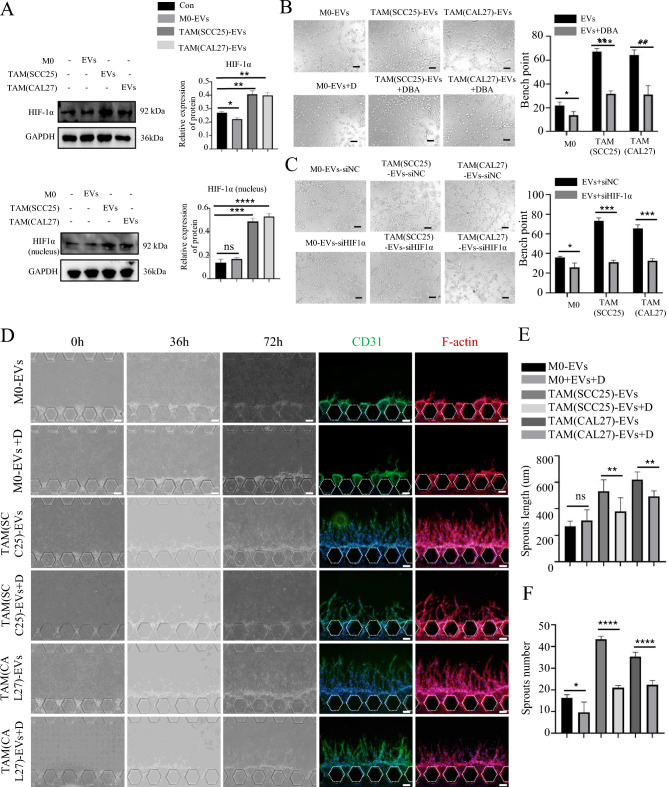
TAM-EVs promote angiogenesis by enhancing HIF-1α level in HUVECs. **A** Protein level of total HIF-1α and nuclear HIF-1α after 72 h incubation of HUVECs with M0/TAM-sEVs. **B** Tube formation assay of HUVECs was induced by M0/TAM-sEVs with/without dimethyl-bisphenol A (DBA, HIF-1α inhibitor, 50 μM) and the numbers of branch points were calculated by ImageJ, Scale bars = 20 μm. **C** Tube formation assay of HUVECs was induced by M0/TAM-sEVs with/without knock-down HIF1α, and the numbers of branch points were calculated by ImageJ, Scale bars = 20 μm. **D** Images of vascular sprouts induced by TAM-sEVs with/without DBA in Microfluidic Chip, Scale bars = 100 μm. **E**,**F** Quantitative analyses of angiogenesis in terms of average sprout length and number. ns > 0.05, * *p* < 0.05, ** *p* < 0.01, *** *p* < 0.001, **** *p* < 0.0001

To investigate whether TAM-sEVs can activate the HIF-1α signaling pathway in ECs, we conducted subsequent validation by western blotting. Our findings revealed that under the induction of TAM-sEVs, there was a substantial rise in the expression of HIF-1α in the nucleus and total cell of HUVECs. In contrast, M0-sEVs exhibited little effect on HIF-1α expression (Fig. 5A). Next, we employed DBA (HIF-1α inhibitor) to test its effects on HUVECs. DBA effectively inhibited tube formation induced by TAM-sEVs (Fig. 5B). Moreover, the tube formation assay also showed that HIF-1α knocking down in HUVECs was significantly suppressed the promoting effect of TAM-EVs on ECs, but the suppressing effect was not obvious in the M0-EVs group (Fig. 5C). This inhibitory effect was further confirmed in the microfluidic chip, where DBA successfully attenuated the length and number of vessel sprouts induced by TAM-sEVs (Fig. 5D–F). These findings provided compelling evidence that HIF-1α served as a crucial factor in TAM-sEV-mediated angiogenesis in HNSCC.

### TAM-EVs upregulated HIF-1α in HUVECs by transferring miR-21-5p

**Fig. 6:**
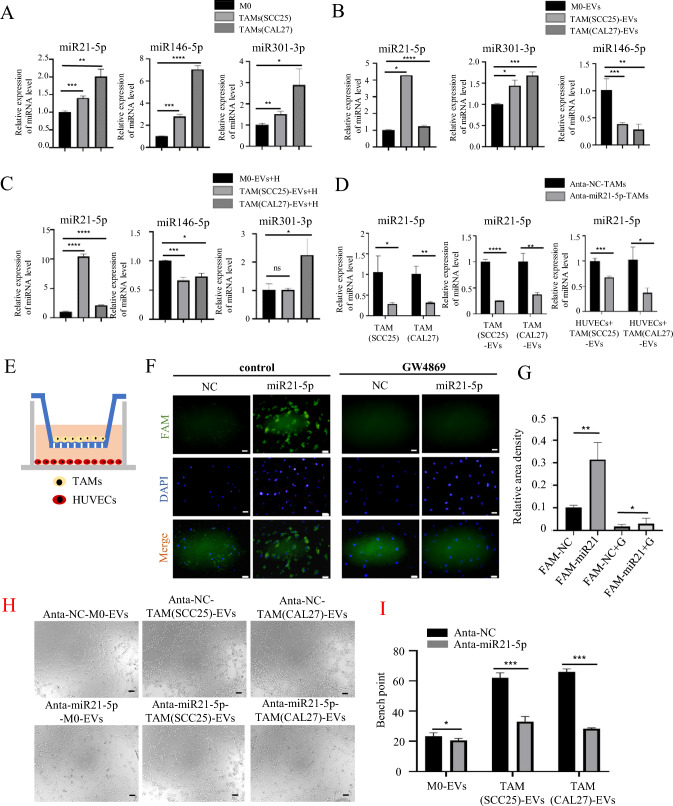
TAM-EVs promote angiogenesis of HUVECs through miR21-5p. **A** RT-qPCR was performed to detect the relative miRNA level in M0 and TAMs. **B** Relative level of miRNA in M0/TAM-sEVs. **C** Relative level of miRNA in HUVECs treated with M0/TAM-sEVs. **D** Relative level of mi21-5p in TAMs, TAM-sEVs and HUVECs induced by TAM-sEVs. **E** Schematic illustration of the in vitro co-culture system. **F** Immunofluorescence images show the Fam-miR21-5p in HUVECs. Scale bars = 50 μm. **G** Average fluorescence intensity of Fam-miR21-5p. **H** Tube formation assay of HUVECs induced by Anta-NC-M0/TAM-sEVs or Anta-miR21-M0/TAM-sEVs, Scale bars = 20 μm. **I** The number of branch points was calculated by ImageJ. * *p* < 0.05, ** *p* < 0.01, *** *p* < 0.001, **** *p* < 0.0001

Extensive studies have consistently shown that EVs are rich in miRNAs [8]. To investigate which specific miRNAs derived from TAM-sEVs are transferred to HUVECs and exert functional effects, we identified three miRNAs, namely, miR-21-5p, miR-146-5p, and miR-301-3p, which are highly expressed in M2 macrophages and have been associated with the activation of the HIF-1α signaling pathway [33–36]. We performed qPCR to examine the expression levels of miR-21-5p, miR-146-5p, and miR-301-3p in M0/TAMs and M0-sEVs/TAM-sEVs (Fig. 6A, B). The results showed significant upregulation of miR-21-5p and miR-146-5p in TAMs and TAM-sEVs compared to that in M0 and M0-sEVs. We further examined the changes in the expression of these three miRNAs in HUVECs after incubation with M0-sEVs/TAM-sEVs for 24 h (Fig. 6C). The results indicated that only the expression of miR-21-5p in HUVECs was significantly increased after incubation with TAM-sEVs compared to the M0-sEVs group. Next, to determine the increased miR-21-5p in ECs derived from TAM-EVs plays a greater role compared to the endogenous miR-21-5p in ECs, RT-qPCR was used to detect the expression of miR21-5p in TAMs, TAM-EVs and HUVECs induced by TAM-EVs with or without miR21-5p. The results showed that in the Anta-miR21-5p-TAMs group, the expression of miR21-5p in TAM-EVs significantly reduced compared to that in Anta-NC-TAMs group. And after HUVECs was treated with Anta-miR21-5p-TAM-EVs, the expression of miR21-5p in HUVECs also decreased remarkably, suggesting that the increased miR21-5p in ECs might be mostly derived from TAM-EVs rather than EC-endogenous miR21-5p (Fig. 6D). Therefore, we speculate that TAMs transfer miR-21-5p to HUVECs through sEVs, thereby activating the HIF-1α signaling pathway to promote HNSCC angiogenesis.

To confirm the transfer of miR-21-5p from TAMs to HUVECs via sEVs, TAMs were transfected with Fam-labeled miR-21-5p and co-cultured with HUVECs for 48 h in a 0.4 μm transwell chamber (Fig. 6E). FAM-positive HUVECs were identified by immunofluorescence microscopy. The results demonstrated that the fluorescence intensity in the control group was much higher than that in the GW4869 (EV inhibitor) group, implying that mi-R21-5p was transferred from TAM-EVs into ECs (Fig. 6F, G). Next, we transfected HUVECs with a miR21-5p inhibitor or miR-21-5p mimic to downregulate or upregulate miR21-5p expression, respectively (Fig. S4A, B). Based on the results of the tube formation assay, the miR-21-5p inhibitor group demonstrated a significant decrease in tube formation compared to the NC inhibitor group. Conversely, the miR21-5p mimic group exhibited a noticeable increase in tube formation compared to the NC mimic group (Fig. S4C–F). Next, we seeded pHUVECs transfected with the miR-21-5p inhibitor in the C4 channel of the microfluidic chip. After 48 h of TAM-sEV induction in the C2 channel, the length and quantity of the sprouts were measured. The miR21-5p inhibitor group had shorter lengths and fewer sprouts than those of the NC inhibitor group (Fig. S4G, H). Furthermore, to test whether the miR21-5p from TAM-EVs enhancing the angiogenic ability of HUVECs, we performed the tube formation assay. The results showed that the Anta-miR21-5p-TAM-EVs on HUVECs significantly reduced the tubular ability of ECs compared to the Anta-NC-TAM-EVs group. While the difference between the Anta-NC-M0-EVs and Anta-miR21-5p-M0-EVs group was not significant (Fig. 6H, I). This suggests that miR-21-5p is transferred from TAMs to HUVECs through sEVs, boosting the angiogenic capability of HUVECs.

### miR21-5p carried by TAM-EVs promoted angiogenesis of HUVECs by activating LATS1/YAP1 and VHL/HIF-1α signaling pathways

**Fig. 7:**
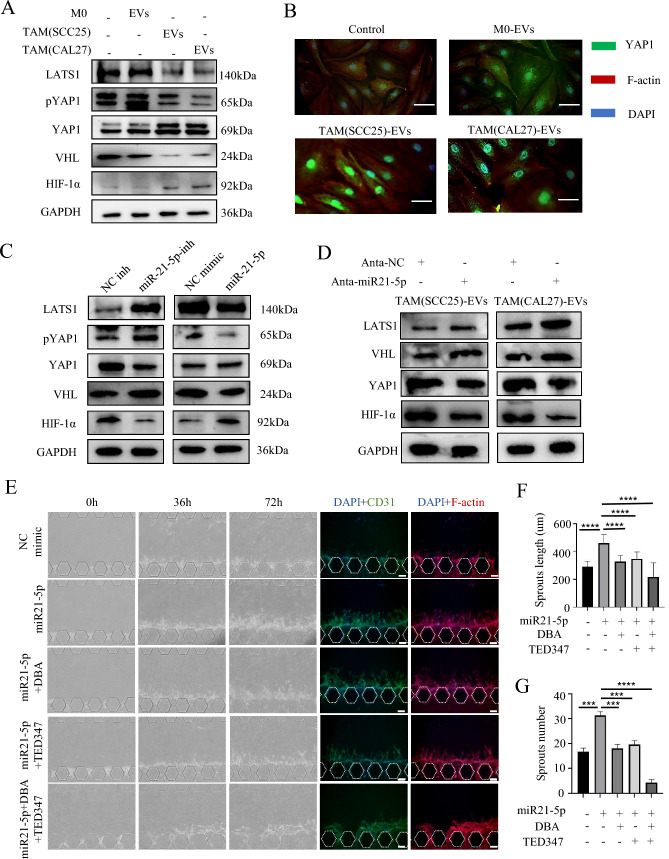
TAM-EVs transfer the miR21-5p to promote angiogenesis through activating the VHL/HIF-1α and LATS1/YAP1/HIF-1α pathway. **A** Protein levels after 24 h incubation of HUVECs with M0/TAM-sEVs. **B** Representative images of IF staining for YAP1 and F-actin in HUVECs after incubation with M0/TAM-sEVs for 24 h. **C** Protein levels in HUVECs after transfection with the NC-inhibitor, miR21-5p inhibitor, the NC-mimic and miR21-5p mimic for 48 h. **D** Protein levels in HUVECs after treatment with M0/TAM-EVs for 48 h that inhibited miR21-5P in M0/TAMs. **E** Microfluidic chips images of vascular sprouts induced by TAM-sEVs with or without DBA/TED347 after transfection with miR21-5p mimic for 24 h, Scale bars = 100 μm. **F**,**G** Quantitative analyses of angiogenesis in terms of average sprout length and number. ** *p* < 0.01, *** *p* < 0.001, **** *p* < 0.0001

To investigate the underlying mechanism of miR-21-5p in HUVECs, we employed an online tool (www.targetscan.org) to predict its interaction with target genes involved in HIF-1α regulation. The analysis revealed that miR-21-5p has a putative binding site in the 3′ untranslated regions (UTRs) of VHL and LATS1 mRNA (Table S2). After treating HUVECs with M0/TAM-sEVs for 24 h, we performed western blotting and RT-qPCR to reveal that TAM-sEVs could decrease LATS1 and pYAP1 expression and increase YAP1 and HIF-1α expression in HUVECs (Fig. 7A; Fig. S5A, B). Subcellular localization of YAP1 by IF staining revealed that TAM-sEVs could increase the nuclear translocation of YAP1 in HUVECs, which suggested that TAM-sEVs might facilitate the dephosphorylation of YAP1, allowing it to translocate into the nucleus, further enhancing HIF-1α transcription (Fig. 7B). Meanwhile, western blotting and RT-qPCR showed that TAM-sEVs could downregulate VHL expression and directly alleviate HIF-1α degradation.

Subsequently, we transfected miR21-5p inhibitor or miR21-5p mimic into HUVECs. Western blotting and RT-qPCR analysis demonstrated that the expression of LATS1 increased in the miR21-5p inhibitor group, along with an elevation in pYAP1, whereas the dephosphorylation level of YAP1 decreased compared to that in the NC group. The results also revealed an upregulation of VHL expression and a decrease in HIF-1α expression in HUVECs treated with miR21-5p inhibitor. The miR21-5p mimic significantly reversed this trend in the HUVECs (Fig. 7C; Fig. S5C, D). Next, we detected the expression of VHL/LATS1/YAP1/HIF1 in HUVECs with the treatment of EVs extracted from TAMs that inhibited miR21-5p. The results showed that compared with the Anta-NC-TAM-EVs group, the expression of VHL and LATS1 in HUVECs were significantly increased in the Anta-miR21-5p-TAM-EVs group, while the expression of YAP1 and HIF-1α was noticeably decreased, suggesting that TAM-EVs could regulate the expression of VHL/LATS1/YAP1/HIF-1α by transmitting miR21-5p to ECs (Fig. 7D; Fig. S5E, F). Finally, HUVECs were transfected with miR21-5p mimic and then treated with YAP1 inhibitor (TED347, 5 μM) and HIF-1α inhibitor (DBA, 50 μM). The results indicated that both TED347 and DBA inhibited the tube-formation ability of HUVECs (Fig. S5G, H). In the microfluidic chip, we found that TED347 and DBA inhibited the length and number of sprouts in pHUVECs and that the combination of the two inhibitors showed a more significant inhibitory effect (Fig. 7E–G). These results collectively indicated that miR21-5p carried by TAM-sEVs activated both the LATS1/YAP1/HIF-1α and VHL/HIF-1α signaling pathways, thereby promoting angiogenesis in HUVECs.

### Targeting miR-21-5p from TAM-EVs inhibited tumor angiogenesis and growth through the YAP1/HIF-1α axis in vivo

**Fig. 8:**
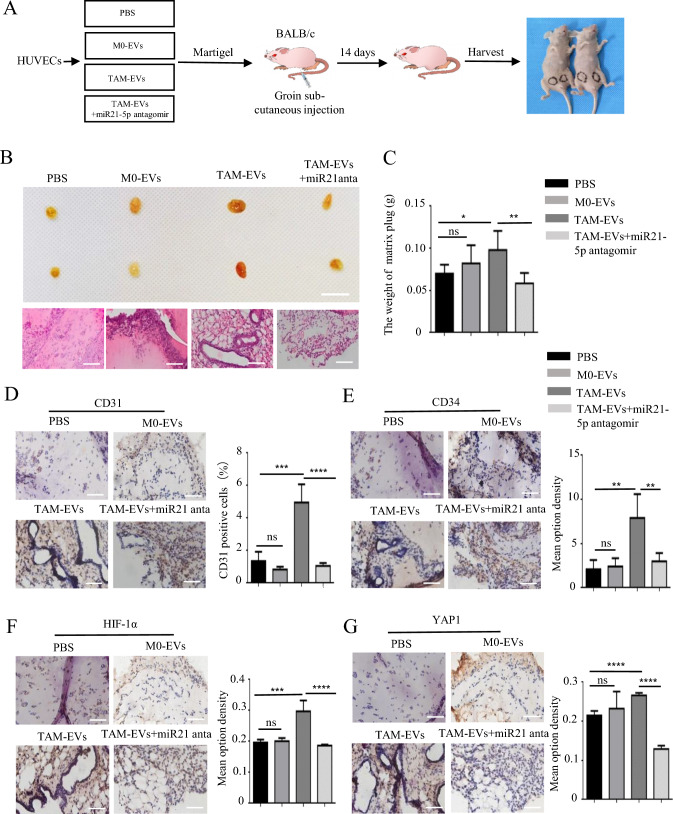
Inhibiting miR21-5p alleviated TAM-EVs induced angiogenesis in matrigel plug angiogenesis models of nude mice. **A** Graphic scheme describing the angiogenesis experiment of matrigel plug assay in vivo. **B** Representative photographs of harvested matrigel plugs and images of H&E staining. Scale bars = 1 cm. **C** The weight of matrigel plugs in each group. **D**–**G** IHC staining and quantitative analysis of CD31, CD34, HIF-1α and YAP1 in each group. ns > 0.05, * *p* < 0.05, ** *p* < 0.01, *** *p* < 0.001

To evaluate the function of the exosomes secreted by TAMs in vivo, we conducted a matrix plug assay in BALB/c nude mice (Fig. 8A). M0/TAM-EVs pretreated with or without miR21-antagomir was mixed with the equal amounts of Matrigel and injected into the groin. After 14 days, the Matrigel plugs were removed and histological staining was performed (Fig. 8B, C). The immunohistochemistry staining results of CD31 and CD34 showed that TAM-sEVs promoted the angiogenic effect of HUVECs in the matrix plug compared to M0-EVs and PBS, whereas inhibiting miR-21-5p alleviated these TAM-sEV effects (Fig. 8D, E). Next, we detected the YAP1 and HIF-1α levels using immunohistochemistry staining and confirmed that TAM-sEVs significantly upregulated YAP1 and HIF-1α expression in the matrix plug, and inhibition of miR-21-5p also could reverse the TAM-sEVs enhancement in angiogenesis (Fig. 8F, G).

**Fig. 9:**
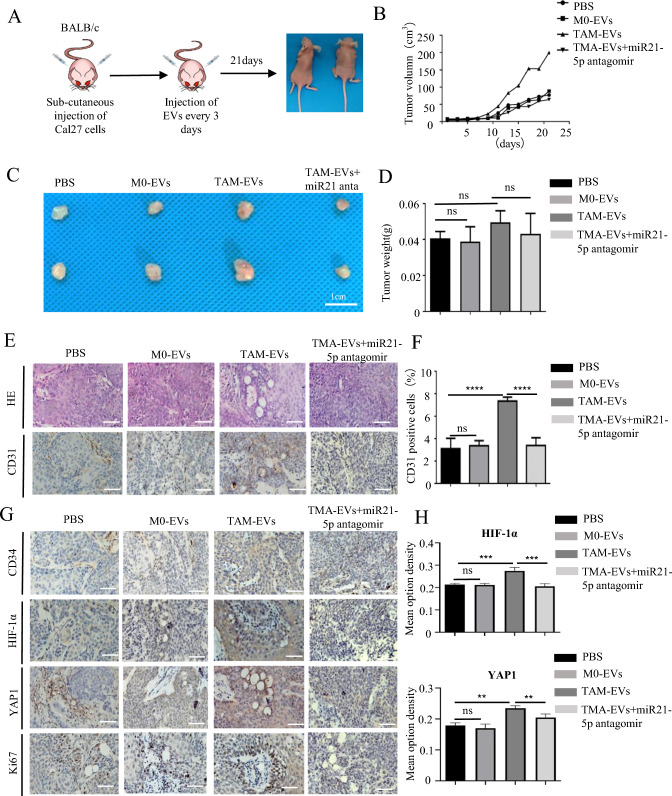
Inhibiting miR21-5p attenuated TAM-EVs induced tumor growth and angiogenesis of HNSCC in vivo. **A** Schematic model of Nude Mice Xenografts. **B** The tumor growth curve shows the tumor size measured every 2 days. **C** Tumor image of each group. **D** The weight of tumors in each group. **E** Representative HE and images of CD31 in tumor tissues. **F** Statistical analysis of CD31 for MVD in each group. **G** Representative IHC staining of CD34, HIF-1α, YAP1, KI67 images in tumor tissues. **H** Statistical analysis of IHC staining for HIF-1α and YAP1 in each group. * *p* < 0.05, ** *p* < 0.01, *** *p* < 0.001

**Fig. 10:**
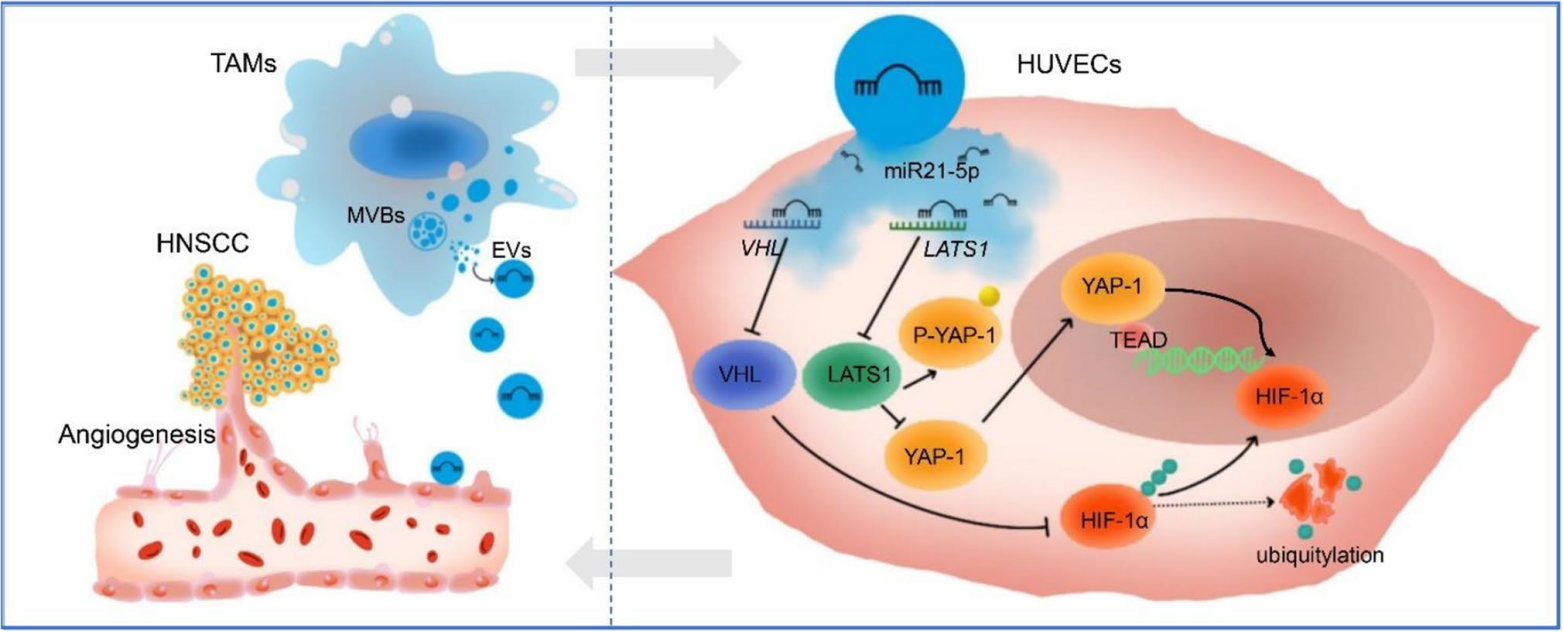
Graphic abstract of TAMs-EVs delivering miR21-5p to endothelial cells to promote angiogenesis and growth via activating the YAP1/HIF-1α axis in HNSCC

To investigate whether TAM-sEVs induced HNSCC growth and angiogenesis, Cal27 cells were subcutaneously injected into nude mice and treated with M0-sEVs and TAM-sEVs every 3 days, as shown in Fig. 9A. The growth kinetics of each subgroup of tumors were measured every 2 days (Fig. 9B). After 21 days, the tumor grafts were harvested. As shown in Fig. 9C and D, the weights were significantly higher in the TAM-sEVs group than in the M0-sEVs and control groups, whereas the weights in the miR21-antagomir group were lower than those in the TAM-sEV group. We measured MVD by staining tumor vascular ECs with CD31 antibodies. Tumors treated with TAM-sEVs showed a significant increase in MVD compared to those treated with M0-sEVs or PBS (Fig. 9E, F), indicating tumor angiogenesis induced by TAM-EVs was positive correlated with tumor growth. Meanwhile, immunohistochemistry results showed that TAM-sEVs increased the expression of CD34, YAP1, HIF-1α, and Ki67, and the inhibition of miR21-5p significantly weakened this strength (Fig. 9G, H). These results collectively suggested that TAM-sEVs from HNSCC promoted tumor growth and angiogenesis in vivo, and miR-21-5p represents a promising therapeutic target for intervention (Fig. 10).

## Discussion

HNSCC progresses from avascular carcinoma in situ and relies heavily on the supply of oxygen and nutrients from nearby blood vessels. When newly formed blood vessels enter the tumor tissue and connect to the bloodstream, the tumor continues to grow, undergoes distant metastasis, and even spreads throughout the body [37–39]. The most HNSCC cases is closely linked to inflammation, which involves the participation of activated macrophages. The quantity of macrophage infiltration serves as a predictive indicator for HNSCC prognosis [40]. In our study, we observed a positive correlation between the quantity of macrophage infiltration and CD31 levels in patients with HNSCC. Additionally, the analysis of TCGA public data revealed a significant positive correlation between TAMs and MVD markers in 520 HNSCC cases. These results suggested that TAMs play an important role in promoting tumor angiogenesis in HNSCC. Investigating the mechanisms underlying this process will further identify whether TAMs can serve as potential targets for anti-angiogenic therapies and provide effective strategies for HNSCC treatment.

As the most abundant immune-infiltrating cell population in HNSCC, TAMs can promote tumor angiogenesis by secreting various angiogenic factors [17]. EVs have been recognized as crucial signaling molecules in TME, which has been demonstrated to play an essential role in the cascade amplification response of tumor angiogenesis [10]. EVs derived from mesenchymal stem cells and tumor cells have been found to facilitate angiogenesis [41]. Other research has shown that M2 macrophage-derived EVs transport miR-501-3p to promote angiogenesis in pancreatic ductal adenocarcinoma by upregulating VEGF-A and VEGFR-2 expression [14]. In our study, we observed that ECs were capable of internalizing EVs derived from TAMs. Moreover, TAM-EVs exhibited a significantly greater enhancement of EC angiogenic potential than M0-EVs. This finding provides new evidence that TAM-EVs contribute to tumor development through angiogenesis in HNSCC.

Angiogenesis is a complex and multifaceted process that plays crucial roles in tumor initiation, progression, and metastasis. One of the primary challenges in angiogenesis research is the identification of appropriate methodologies and models to evaluate the influence of various stimuli on angiogenesis. Our study involved the establishment of a microfluidic chip platform designed to examine blood vessel formation. Our findings demonstrated that TAM-EVs effectively enhanced the length and number of ECs sprouting on the microfluidic chip. Microfluidic chips exhibit several notable features, including low energy consumption, high biomimetic properties, dynamic inducibility, and real-time observation capabilities [42]. Microfluidic chips effectively address the limitations of traditional in vivo and in vitro experiments and provide a more reliable and versatile platform for studying angiogenesis. Establishing a perfused lumen is critical for constructing functional vessels in vitro and assessing the maturity of newly formed vessels. Using the microfluidic chip, we successfully used TAM-EVs to construct a perfused blood vessel lumen. The entire process of blood vessel formation, from the initial sprouting to perfusion, was traced. This further validates the effectiveness and reliability of our microfluidic chip as a biomimetic model for studying tumor angiogenesis in vitro.

Research has revealed that poor prognosis of tumors is closely associated with tumor hypoxia, which is attributed to the increased accumulation of cellular debris in hypoxic regions, as well as the infiltration of TAMs into the hypoxic necrotic area [43]. Recent reports have demonstrated that HIF-1α overexpression in TAMs promotes tumor progression and enhances drug resistance [44]. HIF-1α not only regulates the expression of cancer-related genes in TAMs but also induces metabolic changes, driving tumor development even under conditions of nutrient deprivation and hypoxia [45]. It has been proved that HIF‐1α expression in TAMs located within hypoxic regions of tumors is linked to higher levels of VEGF-A [46]. In breast cancer, HIF‐1α plays an essential role in macrophages to promote tumor angiogenesis through ECs co-cultured with wild-type or HIF-1α-knocked out macrophages [47]. In our study, we first applied single-cell annotation analysis to identify the interactions between TAMs and ECs in HNSCC. Notably, HIF-1α exhibited elevated activity in both TAMs and ECs. Subsequently, we observed that TAM-EVs significantly upregulate the level of HIF-1α in HUVECs. When the expression of HIF-1α was inhibited, the pro-angiogenic effects of TAM-EVs were diminished. These findings suggested that TAM-EVs might promote HNSCC angiogenesis through the HIF-1α pathway.

EVs are abundantly enriched in miRNAs, and hsa-miR-21-5p is a typical onco-miRNA that regulates various cancer-related target genes [48]. Studies have shown that METTL3 contributes to the maturation of miR-21-5p in gestational choriocarcinoma. The increased miR-21-5p subsequently degrades its downstream HIF-1αN (a HIF-1α subunit inhibitor) by targeting its 3′-UTR, leading to the activation of the tumor-promoting HIF-1α/VEGF pathway [49]. Our study showed that TAM-EVs can transfer miR-21-5p to ECs, thereby enhancing tube formation by HUVECs in vitro. Previous studies have demonstrated that miR-21-5p can bind to the 3′-UTRs of *VHL* and *LATS1* mRNA and then increase the level of HIF-1α and YAP1 expression [50, 51]. VHL promotes the ubiquitination of HIF-1α, leading to its degradation and lower expression [52]. Meanwhile, LATS1 exerts its effect on phosphorylating YAP1, which prevents the translocation of YAP1 into the nucleus and its interaction with the transcriptional enhancer factor domain (TEAD), resulting in the suppression of HIF-1α transcription [53]. Recent studies have demonstrated that HIF1α and YAP1 can establish a positive feedback loop, contributing to epithelial-to-mesenchymal transition in pancreatic ductal adenocarcinoma [54]. Our findings revealed that TAM-EVs could downregulate the expression of LATS1 and VHL in HUVECs, inhibiting the phosphorylation of YAP1, increasing the level of HIF-1α and promoting the angiogenic ability of HUVECs. When miR-21-5p was overexpressed in HUVECs, the inhibitors of HIF-1α or YAP1 could significantly decrease the angiogenic capability of HUVECs. Remarkably, this inhibitory effect was most pronounced when both the inhibitors were administered. These findings suggested that TAM-EVs carrying miR-21-5p acted on ECs and simultaneously activated the LATS1/YAP-1/HIF-1α and VHL/HIF-1α signaling pathways, thereby upregulating the level of HIF-1α to enhance the angiogenic potential in HUVECs. In our in vivo experiments, we further confirmed that TAM-EVs enhanced HNSCC angiogenesis and tumor growth through the miR-21-5p/YAP1/HIF-1α axis. However, the targeting of TAM-EVs and the specific mechanisms by which HIF-1α regulates angiogenesis in HNSCC warrant further investigation.

## Conclusion

Our study demonstrated that TAMs could transfer EVs to ECs, thereby enhancing their angiogenic capacity both in vitro and in vivo. In terms of the mechanism, we found that miR-21-5p carried by TAM-EVs activated HIF-1α in ECs through VHL and LATS1, forming a YAP1/HIF-1 positive loop, which plays a crucial role in promoting tumor angiogenesis. We successfully established a robust microfluidic chip platform for studying HNSCC angiogenesis and efficiently induced functional and mature vascular lumens, thereby providing a reliable in vitro model for investigating tumor-associated angiogenesis. This study improves our understanding of the molecular mechanism of TAM-EVs in tumor angiogenesis and identifies the miR-21-5p/YAP1/HIF-1 axis as a potential therapeutic target for HNSCC.

### Supplementary Information

Below is the link to the electronic supplementary material.

Supplementary file1 (PDF 174 KB)

Supplementary file2 (PDF 271 KB)

Supplementary file3 (DOCX 1723 KB)

## Data Availability

All software and algorithms used in this study were obtained from free or commercially available and listed in the “Methods” section. All data generated or analyzed during this study are included in this published article [and its Additional files].

## Abbreviations

EC: Endothelial cell
EV: Extracellular vesicle
sEV: Small extracellular vesicle
HUVEC: Human umbilical vein endothelial cell
pHUVEC: Pulmonary HUVEC
MVD: Microvessel density
HNSCC: Head and neck squamous cell carcinoma
TAM: Tumor-associated macrophage
TME: Tumor microenvironment
TAM-EV: TAM-derived EV
HIF-1α: Hypoxia-inducible factor 1-alpha
VEGF: Vascular endothelial growth factor
NF: Normal human fibroblast
NTA: Nanoparticle tracking analysis
DMEM: Dulbecco’s modified Eagle medium
ECM: EC medium
RT-qPCR: Reverse transcription quantitative real-time PCR
IOD: Integrated optical density
PDMS: Polydimethylsiloxane
PCA: Principal component analysis
t-SNE: T-distributed neighbor embedding
FDR: False discovery rate
UTR: Untranslated region
