# Supplementary figures and images for: Tumor-associated macrophage-derived exosomal miR21-5p promotes tumor angiogenesis by regulating YAP1/HIF-1α axis in head and neck squamous cell carcinoma
Source: Cell Mol Life Sci. 2024 Apr 11;81(1):179. doi: 10.1007/s00018-024-05210-6 (PMC11009780; doi:10.1007/s00018-024-05210-6)

Fig. 2C

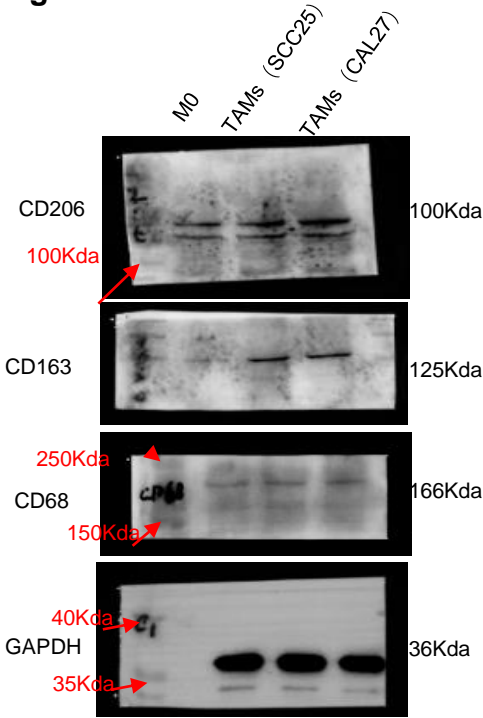

Fig. 2G

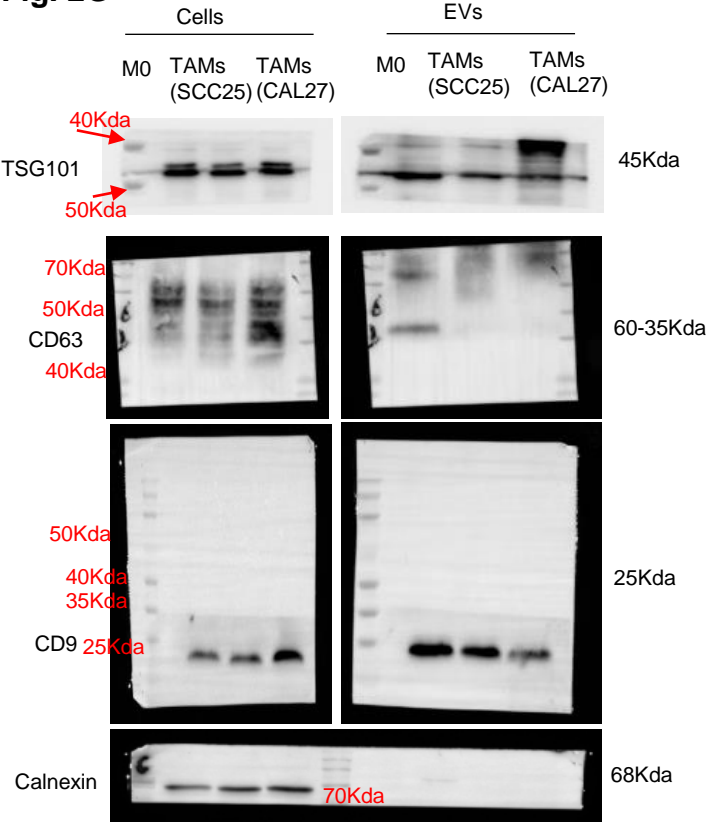

Fig. 5A

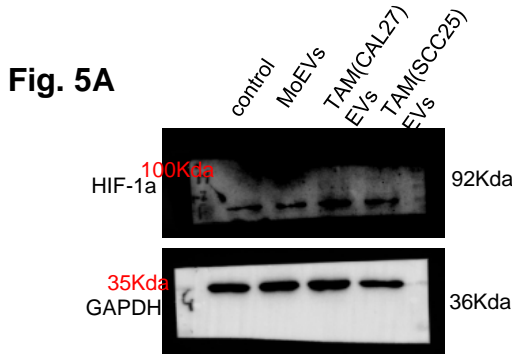

Fig. 7A

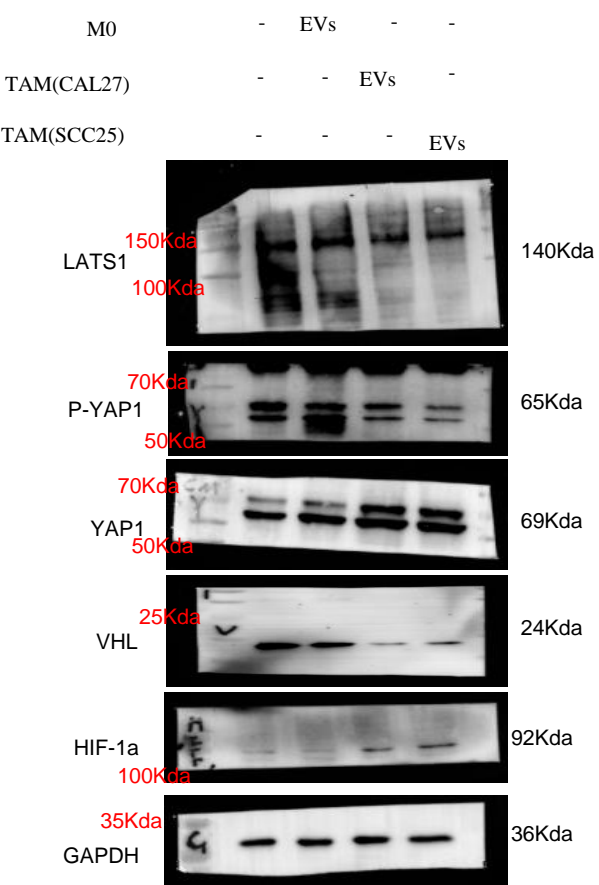

Fig. 7C

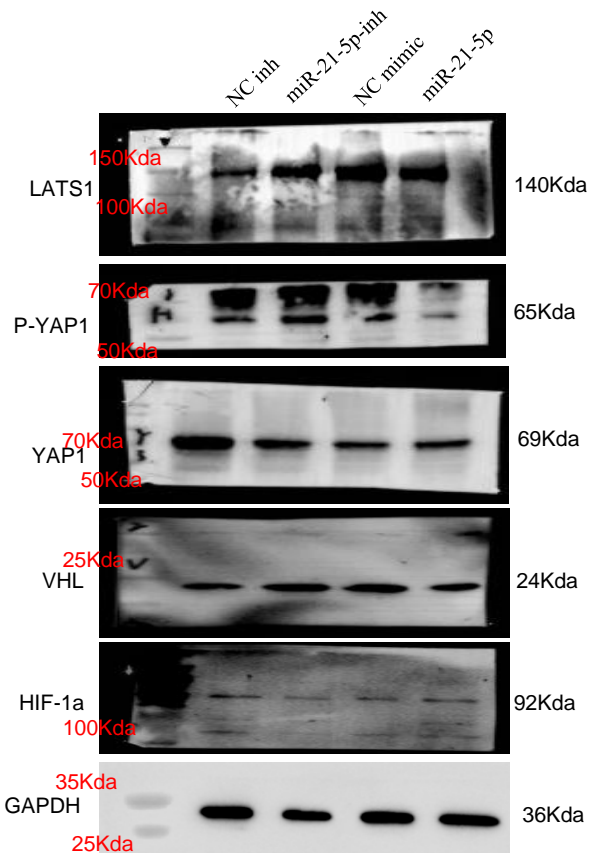

Supplement: Supplementary file 1 — Supplementary file1 (PDF 174 KB) [file 18_2024_5210_MOESM1_ESM.pdf]

Figure 2F

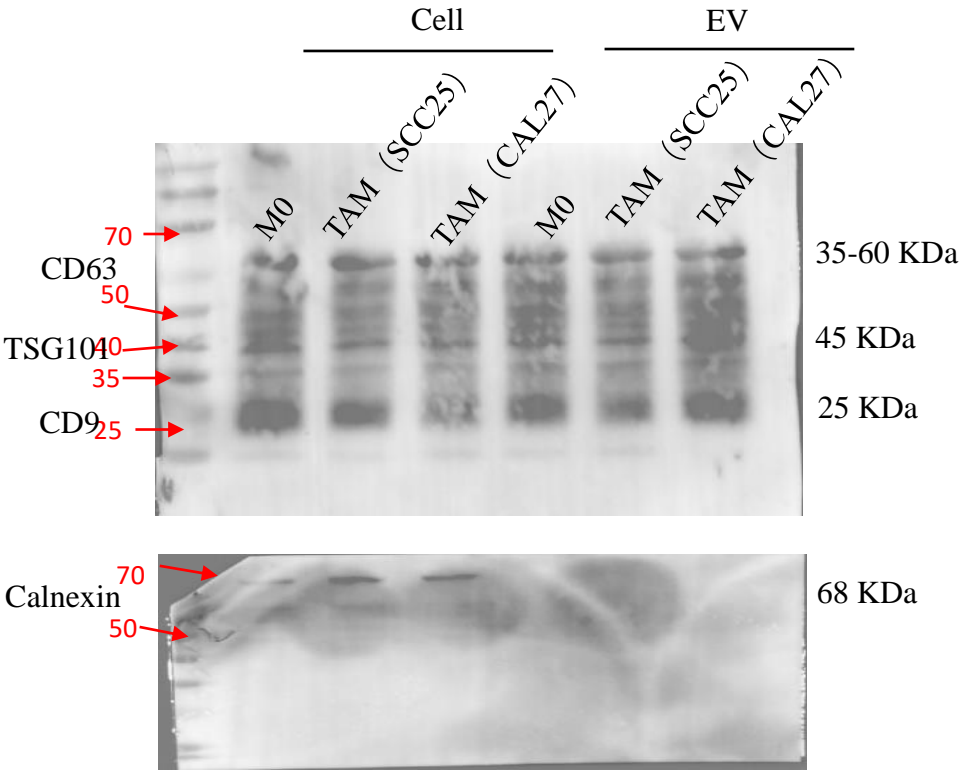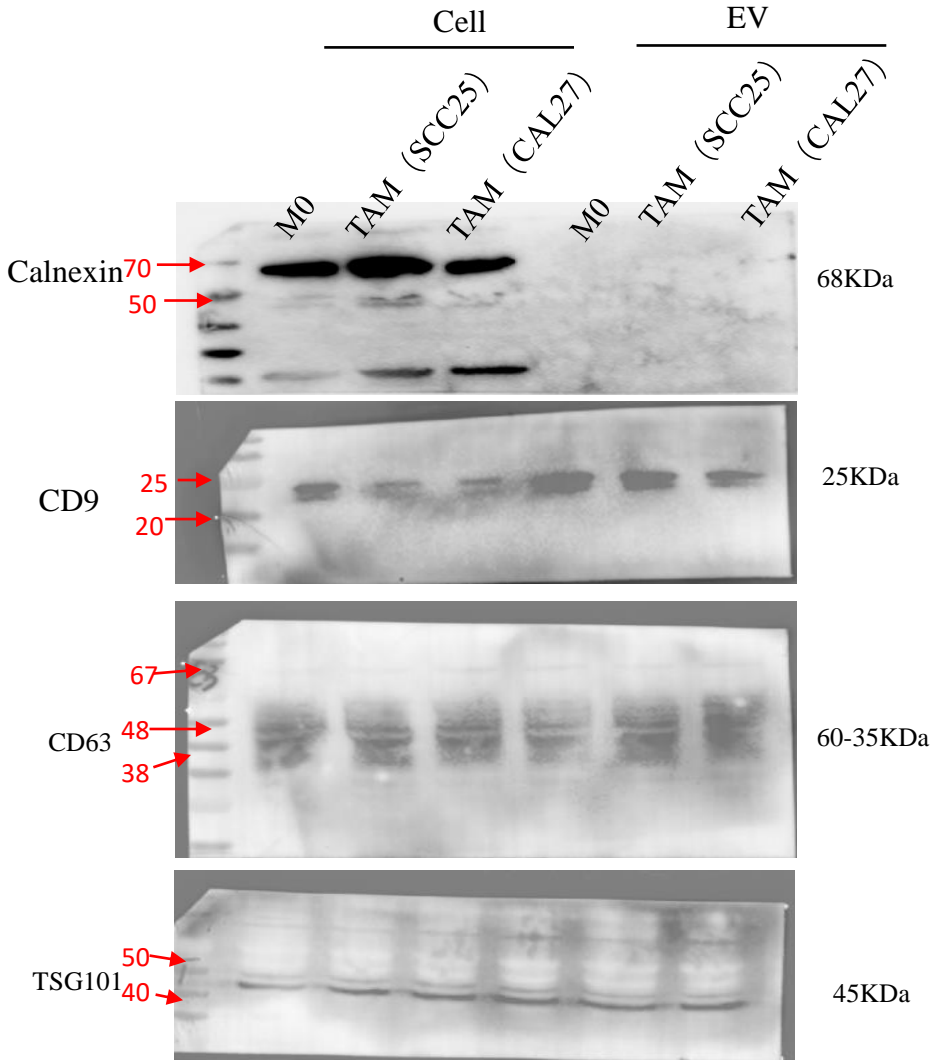

Figure 5A

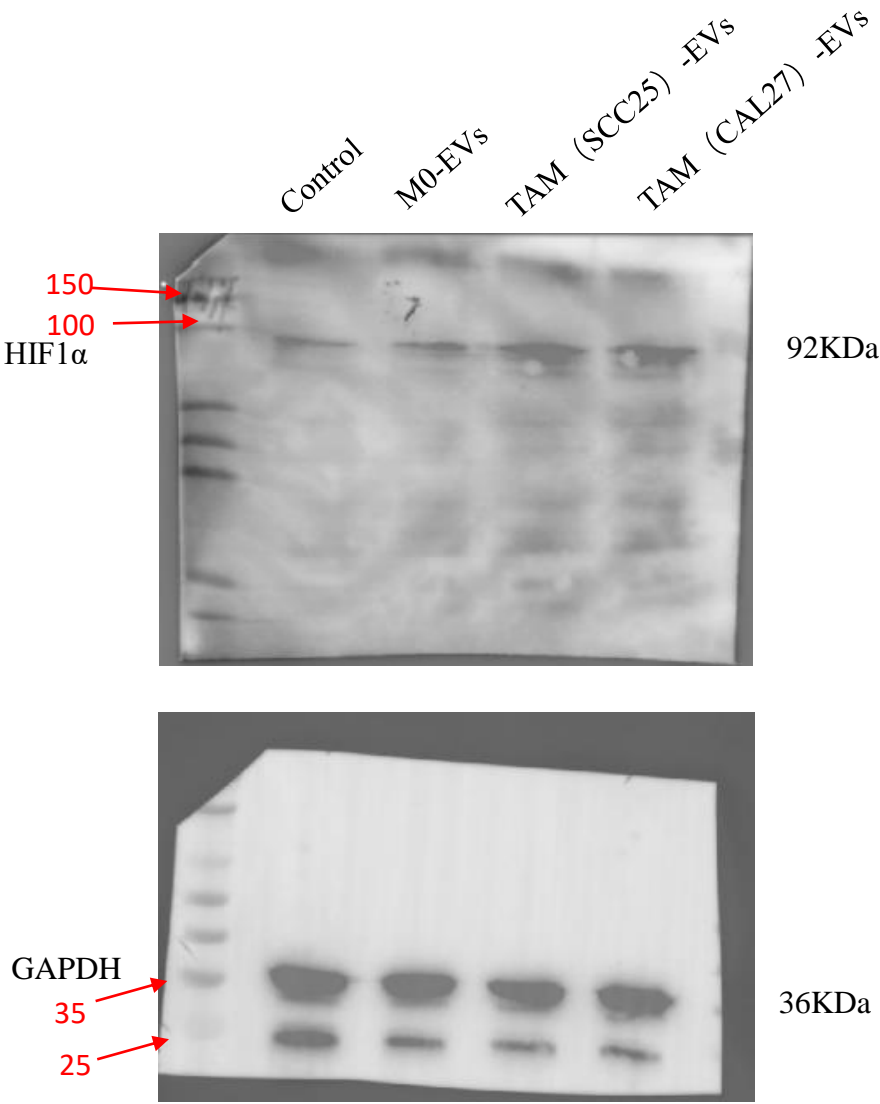

Figure 7D

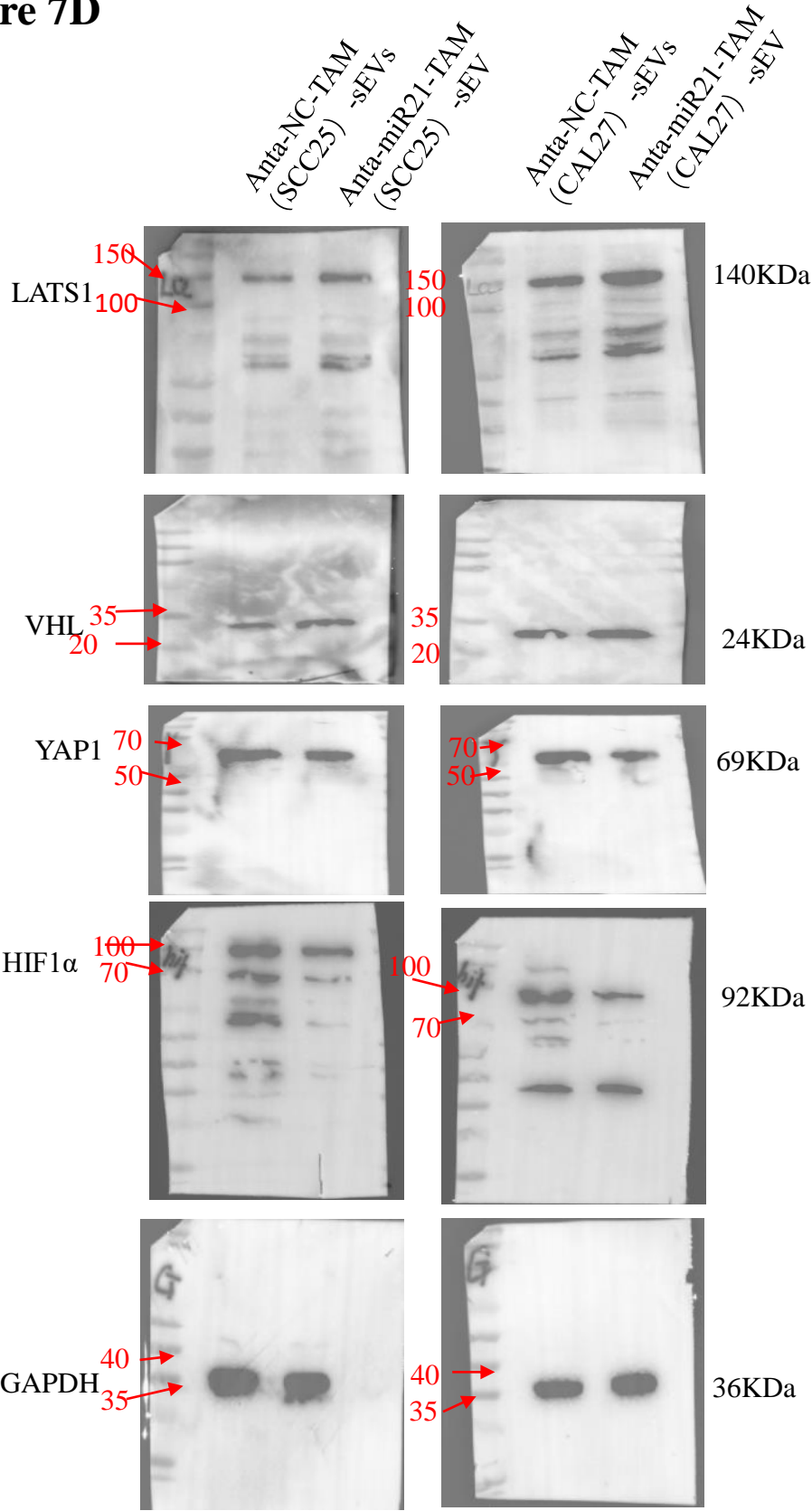

Supplement: Supplementary file 2 — Supplementary file2 (PDF 271 KB) [file 18_2024_5210_MOESM2_ESM.pdf]
