# Supplementary material for: Tumor-associated macrophage-derived exosomal miR21-5p promotes tumor angiogenesis by regulating YAP1/HIF-1α axis in head and neck squamous cell carcinoma
Source: Cell Mol Life Sci. 2024 Apr 11;81(1):179. doi: 10.1007/s00018-024-05210-6 (PMC11009780; doi:10.1007/s00018-024-05210-6)
Supplement: Supplementary file 3 — Supplementary file3 (DOCX 1723 KB) [file 18_2024_5210_MOESM3_ESM.docx]

**Supplementary Information**

**Supplementary Tables**

**Table S1 Primers for RT-qPCR**

| **Primers** | **The primer sequence (5’- 3’)** |
| --- | --- |
| **hsa-FIZZ1** | F:GCAAGAAGCTCTCGTGTGCTAG  R:AACATCCCACGAACCACAGCCA |
| **hsa-ARG1** | F: TCATCTGGGTGGATGCTCACAC  R: GAGAATCCTGGCACATCGGGAA |
| **hsa-GAPDH** | F: AGCACCGTCAAGGCTGAGAAC  R: TGGTGAAGACGCCAGTGGA |
| **hsa-miR-21-5p** | RT:GTCGTATCCAGTGCAGGGTCCGAGGTATTCGCACTG  GATACGACtcaaca  F: CCCCCTAGCTTATCAGACTGATG |
| **hsa-miR-146a-5p** | RT: GTCGTATCCAGTGCAGGGTCCGAGGTATTCGCACTG  GATACGACaaccca  F: CCCCTGAGAACTGAATTCCATG |
| **hsa-miR-301a-3p** | RT:GTCGTATCCAGTGCAGGGTCCGAGGTATTCGCACTG  GATACGACgctttg  F: CCCCCAGTGCAATAGTATTGTCA |
| **U6** | F: CTCGCTTCGGCAGCACA  R:AACGCTTCACGAATTTGCGT |
| **Universal R** | CCAGTGCAGGGTCCGAGGT |

**Table S2 The sequence for miR-21 targeting genes**

| **Targeting gene** | **LATS1** | **VHL** |
| --- | --- | --- |
| **Position** | 463-470 of LATS1 3' UTR | 463-470 of LATS1 3' UTR |
| **Predicted consequential pairing of target region** | 5'  ...UGUUUUUGGUAGAAAAUAAGCUA... | 5'    ...UUGCUCUAUGUUAGUAUAAGCUU... |
| **Predicted consequential pairing of target miRNA** | 3'      AGUUGUAGUCAGACUAUUCGAU | 3'        AGUUGUAGUCAGACUAUUCGAU |
| **Site type** | 8mer | 7mer-m8 |
| **Context ++ score** | -0.10 | -0.20 |
| **Context ++ score percentile** | 78 | 91 |
| **Weighted context ++ score** | -0.10 | -0.19 |
| **Conserved branch length** | 3.419 | 3.157 |
| **Pct** | 0.50 | 0.20 |
| **Predicted relative KD** | -4.787 | -3.849 |

* The predicted binding sites of miR21-5p with *VHL* and *LATS1* mRNA (https://www.targetscan.org/)

**Supplementary Figure legends and Figures**

**Figure S1**


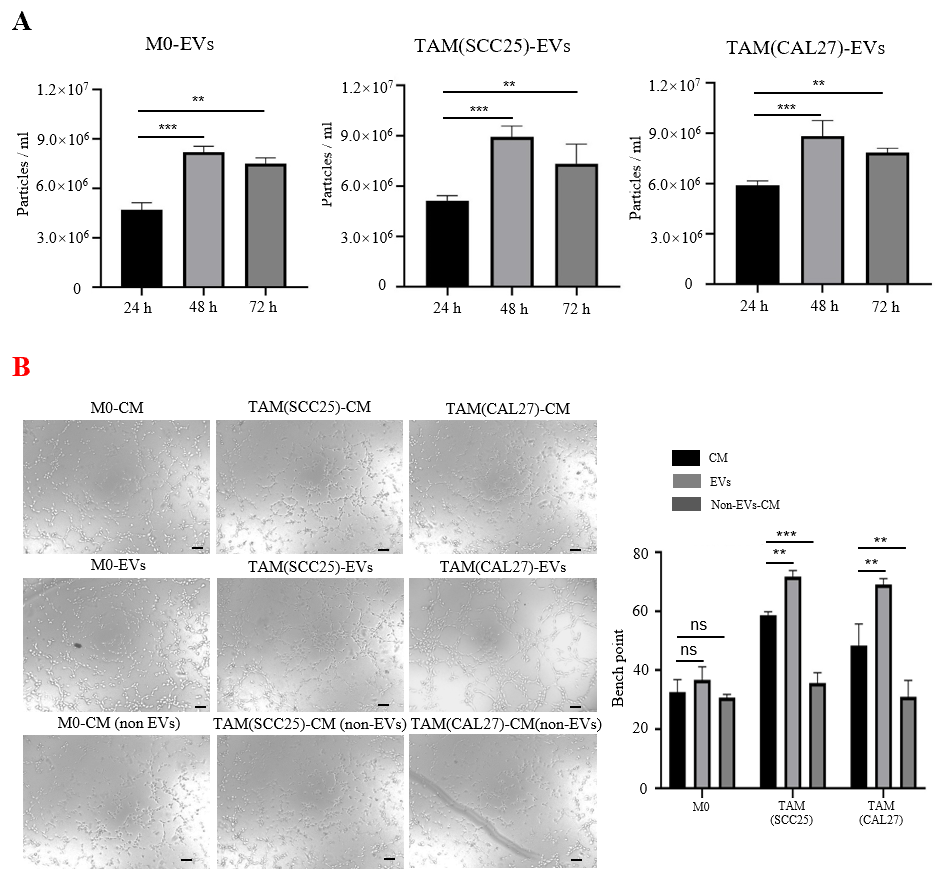


**Figure S1 The EVs derived from TAMs promote the tube formation of ECs. A** NTA analysis of EVs in the conditioned medium of M0/TAMs at different time points.

**B** Representative micrographs of the tube formation assay after treatment of M0/TAM-CM, M0/TAM-EVs and M0/TAM-CM (non-EVs), Scale bars= 20um.

**Figure S2**


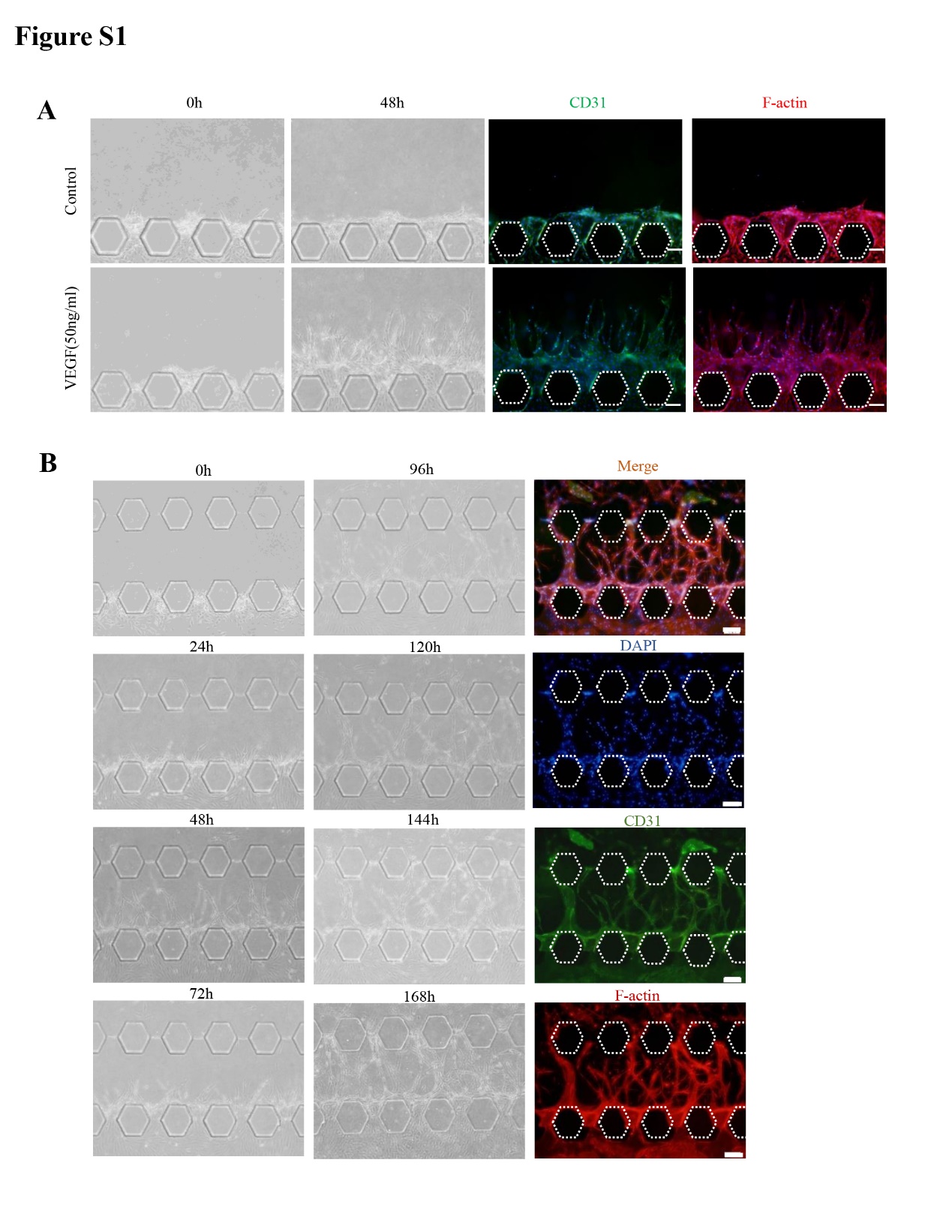


**Figure S2 Establishment and assessment of the microfluidic chip for investigation of angiogenesis**. **A** Images of vascular sprouts induced by VEGF165, Scale bars= 100um. **B** TAM-EVs induced the formation of mature perusable blood vessels in the microfluidic chip. Scale bars = 100um. **B** Physical diagram of a microfluidic chip compared with a one-yuan Chinese currency coin.

**Figure S3**
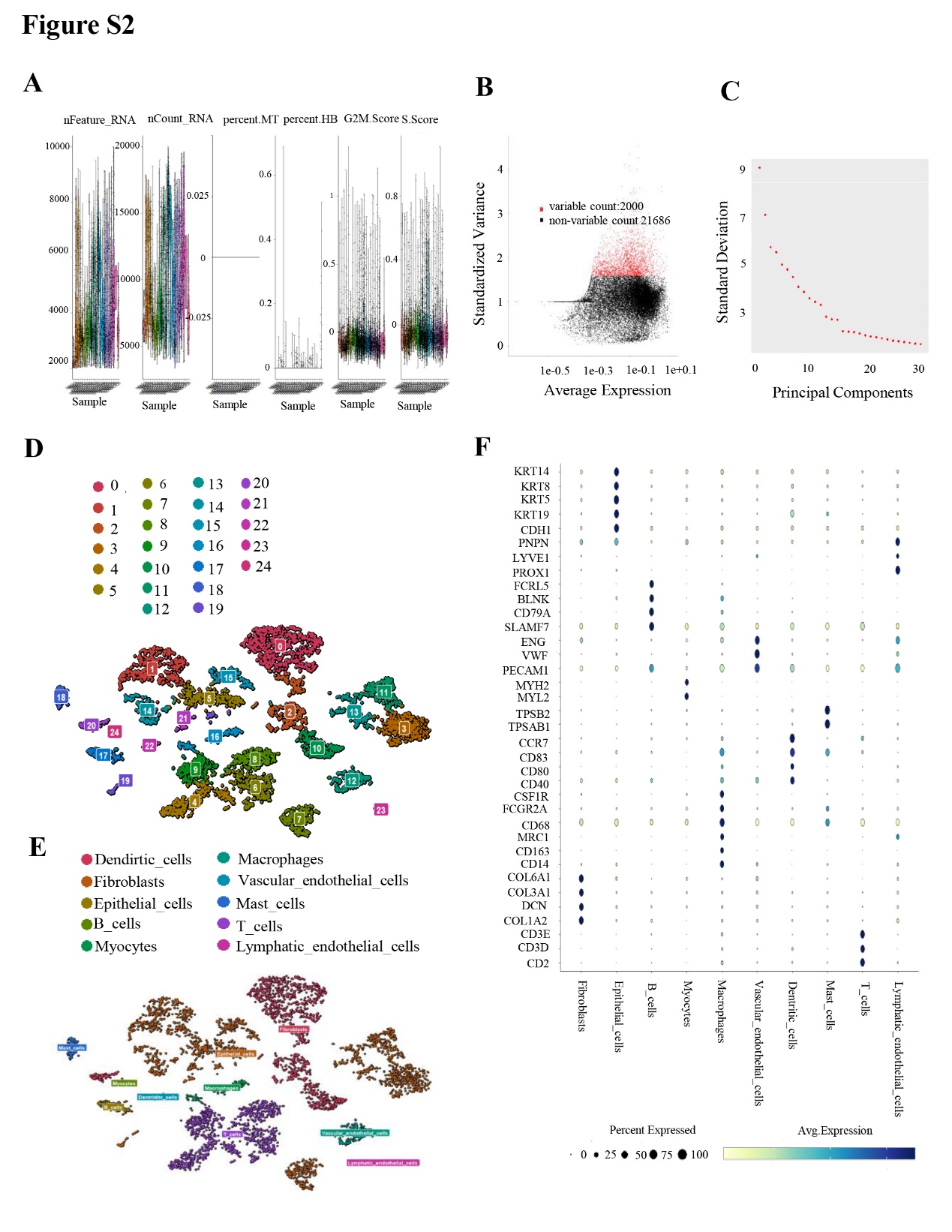


**Figure S3 The single-cell annotation and processing in HNSCC. A** The single-cell sequencing data in HNSCC was quality controlled using R 4.1.3 and the Seurat package, yielding 5886 high-quality cells for further analysis. **B** The variance plot showed the expression levels of 5,886 genes in the dataset. And 2,000 highly variable genes were identified and marked in red. **C** PCA was used to reduce cell dimensionality based on the expression levels of 2,000 highly variable genes. The scatter plot showed standard deviations of the first 30 principal components (PC). **D** Using the top 30 PC, clustering and partitioning were performed on 5,886 cells, and the high-dimensional information was visualized on a two-dimensional plane using the t-SNE algorithm, resulting in 25 cell clusters. **E** By combining SingleR machine annotation with manual correction, these 25 cell clusters were successfully annotated into 10 distinct cell types. **F** The bubble plot displays the hallmark genes of these 10 cell types.

**Figure S4**


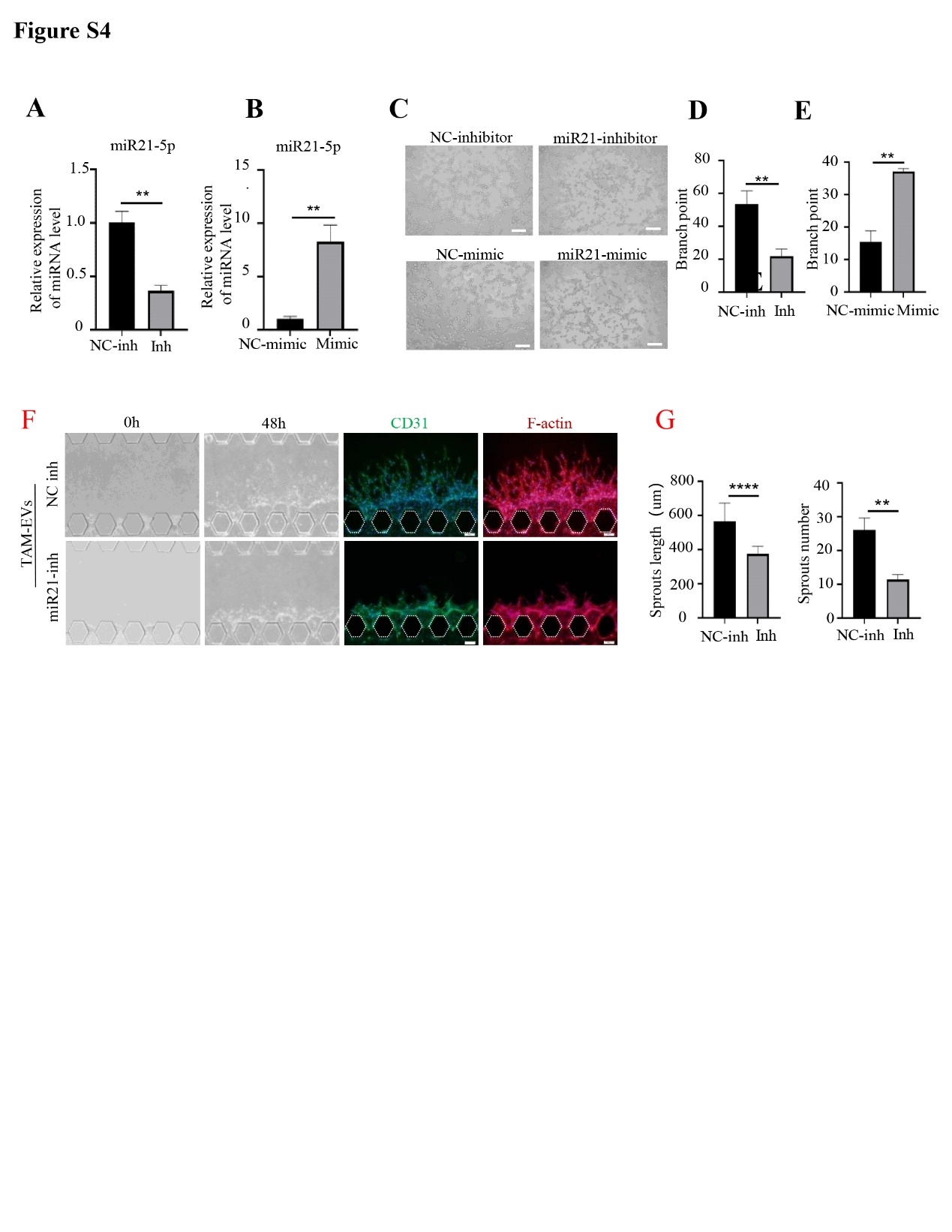


**Figure S4 miR21-5p enhances the ability of tube formation in HUVECs. A** Relative level of miRNA in HUVECs after transfection with miR21-5p inhibitor and the negative control (NC-inh) for 48h. **B** Relative level of miRNA in HUVECs after transfection with miR21-5p mimic and the negative control (NC-mimic) for 48h. **C** Representative micrographs of the tube formation assay after transfection with miR21-5p inhibitor, or miR21-5p mimicfor 48h, Scale bars= 100um. **D and E** The numbers of branch points were calculated by ImageJ. **F** Microfluidic chips images of vascular sprouts after transfection with the NC-inhibitor or miR21-5p inhibitor for 24h , Scale bars= 100um. **G** Quantitative analyses of angiogenesis in terms of average sprout length and number.

**Figure S5**


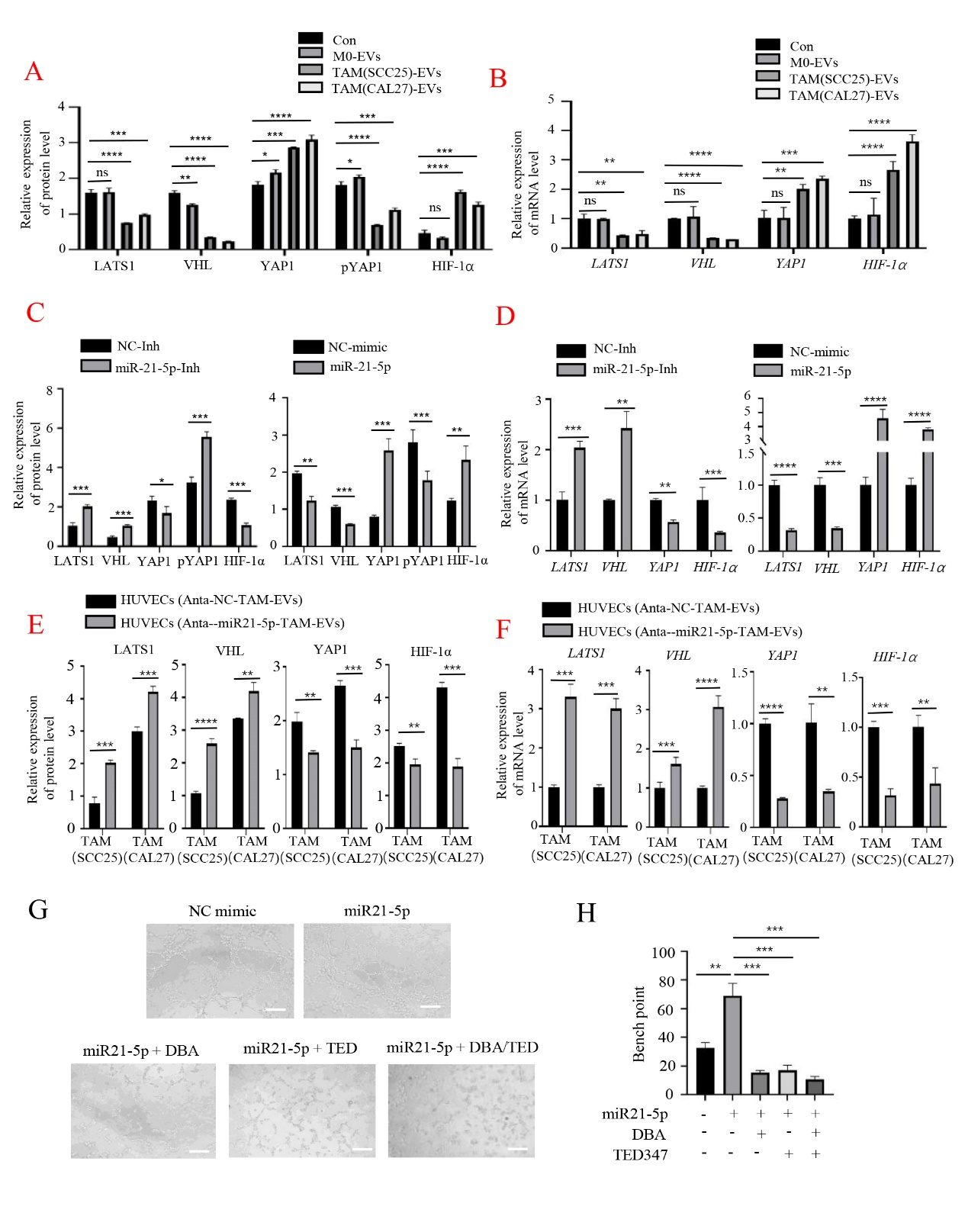


**Figure S5 The miR21-5p carried by TAM-EVs promotes angiogenesis through activating the VHL/HIF-1α and LATS1/YAP1/HIF-1α pathway.** **A** Quantitative analysis of the protein level in HUVECs treated with M0/TAM-EVs. **B** The mRNA expression of HUVECs treated with M0/TAM-EVs was measured by RT-qPCR. **C** Quantitative analysis of the protein level in HUVECs up/down-regulated by miR21-5P. **D** The mRNA expression of HUVECs up/down-regulated by miR21-5P was measured by RT–qPCR. **E** Quantitative analysis of the protein level in HUVECs treated with Anta-miR21-5P-M0/TAM-EVs. **F** The mRNA expression of HUVECs treated with Anta-miR21-5P-M0/TAM-EVs was measured by RT-qPCR. **G** Representative micrographs of the tube formation assay after transfection of miR21-5p with or without DBA (HIF-1α inhibitor)/TED347 (YAP1 inhibitor) for 48h, Scale bars= 100um. **H** The numbers of branch points were calculated by ImageJ.
